# The built-in selection bias of hazard ratios formalized using structural causal models

**DOI:** 10.1007/s10985-024-09617-y

**Published:** 2024-02-15

**Authors:** Richard A. J. Post, Edwin R. van den Heuvel, Hein Putter

**Affiliations:** 1 Department of Mathematics and Computer Science, Eindhoven University of Technology, Eindhoven, The Netherlands; 2 Department of Biomedical Data Sciences, Leiden University Medical Center, Leiden, The Netherlands; 3 Mathematical Institute, Leiden University, Leiden, The Netherlands

**Keywords:** Causal inference, Causal hazard ratio, Selection bias, Individual effect modification, Heterogeneity of treatment effects, 62D20, 62N02

## Abstract

It is known that the hazard ratio lacks a useful causal interpretation. Even for data from a randomized controlled trial, the hazard ratio suffers from so-called built-in selection bias as, over time, the individuals at risk among the exposed and unexposed are no longer exchangeable. In this paper, we formalize how the expectation of the observed hazard ratio evolves and deviates from the causal effect of interest in the presence of heterogeneity of the hazard rate of unexposed individuals (frailty) and heterogeneity in effect (individual modification). For the case of effect heterogeneity, we define the causal hazard ratio. We show that the expected observed hazard ratio equals the ratio of expectations of the latent variables (frailty and modifier) conditionally on survival in the world with and without exposure, respectively. Examples with gamma, inverse Gaussian and compound Poisson distributed frailty and categorical (harming, beneficial or neutral) distributed effect modifiers are presented for illustration. This set of examples shows that an observed hazard ratio with a particular value can arise for all values of the causal hazard ratio. Therefore, the hazard ratio cannot be used as a measure of the causal effect without making untestable assumptions, stressing the importance of using more appropriate estimands, such as contrasts of the survival probabilities.

## Introduction

When interested in time-to-event outcomes, ideally, one would like to know the hazard rates of an individual in the worlds with and without exposure. It is then standard practice to fit the observed hazard rates with a (time-invariant) Cox model (Cox 1972) to estimate the ratio of the expected hazard rates in both worlds. A decade ago, Hernán (2010) raised awareness that hazard ratios estimated from a randomized controlled trial (RCT) are unsuitable for causal inference. Firstly, the average hazard ratio could be uninformative as there will typically be time-varying hazard ratios. More importantly, even when period-specific hazard ratios are estimated, these can vary solely due to the loss of randomization over time by conditioning on survivors. The exposure assignment and risk factors become dependent when conditioning on individuals that survived *t*, i.e. survival time $T{\ge } t$, even if these risk factors are unrelated to the exposure (Aalen et al. 2015). As a result, effect measures based on hazard rates can suffer from non-collapsibility (Martinussen and Vansteelandt 2013; Aalen et al. 2015; Sjölander et al. 2016; Daniel et al. 2021).

In practice, the ratio of (partly) marginalized hazards, is estimated, that by the non-collapsibility, deviates from the conditional (causal) hazard ratio. This contrast is referred to as the built-in selection bias of hazard ratios as the bias results from conditioning on prior survival (Hernán 2010; Aalen et al. 2015; Sjölander et al. 2016; Stensrud et al. 2018; Young et al. 2020; Martinussen et al. 2020). This bias should not be confused with confounding bias that is absent when using data from an RCT (Didelez and Stensrud 2021). For exposure assignment *A*, and the potential survival time when the exposure is intervened on to *a* denoted by $T^{a}$, the expected observed hazard ratio from an RCT satisfies

1 $$\begin{aligned} \frac{\lim _{h\rightarrow 0}h^{-1}{\mathbb {P}}\left( T \in [t,t+h) \mid T{\ge } t, A{=}a \right) }{\lim _{h\rightarrow 0}h^{-1}{\mathbb {P}}\left( T \in [t,t+h) \mid T{\ge } t, A{=}0 \right) } = \frac{\lim _{h\rightarrow 0}h^{-1}{\mathbb {P}}\left( T^{a} \in [t,t+h) \mid T^{a}{\ge } t \right) }{\lim _{h\rightarrow 0}h^{-1}{\mathbb {P}}\left( T^{0} \in [t,t+h) \mid T^{0}{\ge } t \right) }\qquad \end{aligned}$$

(De Neve and Gerds 2020; Martinussen et al. 2020). The expected observed hazard ratio thus equals the ratio of hazard rates at time *t* for the potential outcomes of individuals from different populations; those for which $T^{a}{\ge } t$ and those for which $T^{0}{\ge } t$. As indicated before, these populations will typically not be exchangeable in other risk factors, implying that an effect found cannot be (solely) assigned to the exposure. The effect does thus not reflect how the hazard rate of an individual is affected by exposure. Only for cause-effect relations such that (1) is time-invariant, the estimand can be interpreted as $\frac{\log ({\mathbb {P}}(T^{a}{\ge } t))}{\log ({\mathbb {P}}(T^{0}{\ge } t))}$. It has been recommended to use better interpretable estimands such as contrasts of quantiles, the restricted mean survival or survival probabilities of the potential outcomes respectively (Hernán 2010; Stensrud et al. 2018; Bartlett et al. 2020; Young et al. 2020), or the probabilistic index derived from the latter (De Neve and Gerds 2020). Alternatively, one can avoid interpretation issues by using accelerated failure time models (Hernán et al. 2005; Hernán 2010) or additive hazard models (Aalen et al. 2015; Martinussen et al. 2020).

Nevertheless, particularly in medical sciences, observed hazard ratios are still commonly presented by practitioners. In this paper, we formalize how the expected observed hazard ratio deviates from the causal hazard ratio (as defined in Sect. 2), and thus quantify the built-in selection bias. To do so, we first present a general parameterization of cause-effect relations for time-to-event outcomes using a structural causal model in Sect. 2 and explain the loss of randomization over time by conditioning on survivors. We will limit ourselves to systems where the causal effect is appropriately described by a causal hazard ratio. The quantitative examples in which the hazard under no exposure varies among individuals, i.e. frailty, as presented in the literature (Aalen et al. 2015; Stensrud et al. 2017; Balan and Putter 2020) do fit in our framework, and we will formalize results for these examples. Additionally, we will extend these examples with causal effect heterogeneity, i.e. the causal effect on the hazard rate might vary between individuals (Stensrud et al. 2017). In Sect. 3, we define the causal hazard ratio and explain why this estimand is not identifiable from data. Practioners instead compute the hazard ratio from data, and in Sect. 4 (which comprises the bulk of this paper) we derive what estimand is estimated: the survivor marginalized causal hazard ratio. This estimand describes a combination of the causal effect of interest and the difference in latent frailty- and modifying-features distribution between survivors in the exposed and unexposed universe. We point out exactly how this estimand deviates from the causal hazard ratio in the presence of frailty and effect heterogeneity. To develop understanding of how selection of frailty- and modifying-features affect the value of the estimand, we presented examples for systems in the presence of frailty (Sect. 4.1), effect heterogeneity (Sect. 4.2) or both (Sect. 4.3). In Sect. 5, we shortly discuss the implications of our results for the traditional Cox estimand. Finally, we present some concluding remarks in Sect. 6.

## Notation

In this paper, probability distributions of factual and counterfactual outcomes are defined in terms of the potential outcome framework (Neyman 1990; Rubin 1974). Let $T_{i}$ and $A_{i}$ represent the (factual) stochastic outcome and exposure assignment level of individual *i*. Let $T_{i}^{a}$ equal the potential outcome of individual *i* under an intervention of level *a* (counterfactual when $A_{i} \ne a$). For those more familiar with the do-calculus, $T^{a}$ is equivalent to $T \mid do(A{=}a)$ as e.g. derived in (Pearl 2009, Equation 40) and (Bongers et al. 2021, Definition 8.6). Throughout this paper, we will assume causal consistency: if $A_{i} = a$, then $T_{i}^{a} = T_{i}^{A_{i}} = T_{i}$, implying that potential outcomes are independent of the assigned exposure levels of other individuals.

The hazard rate of $T^{a}$ can vary among the individuals in the population of interest. We will parameterize this heterogeneity for hazards of $T^{0}$ using a random variable $U_{0i}$ that represents the frailty of individual *i* (see for example (Aalen et al. 2008, Chapter 6) or Balan and Putter (2020)). There can also be (relative) effect heterogeneity that we parameterize using the random variable $U_{1i}$, giving rise to an individual-specific hazard ratio. The hazard of the potential outcome $T_{i}^{a}$ can be parameterized with a function that depends on $U_{0i}$, $U_{1i}$ and *a*. We describe cause-effect relations with a structural causal model (SCM) which is commonly used in the causal graphical literature, see e.g. (Pearl 2009, Chapter 1.4) and (Peters et al. 2018, Chapter 6), to model observations. Instead, we include details on individual effect modifier $U_{1}$ as well as the latent common cause of the outcomes $U_{0}$, to describe all the potential outcomes of an individual jointly. A SCM as presented in this paper is therefore a union of the SCM for observations ($A{=}a$), and the so-called intervened SCMs for all possible $do(A{=}a)$. SCMs have been used before to describe hazards of potential outcomes (Hernán et al. 2000, 2001, 2005). Formulation of hazard rates of potential outcomes presented in the literature, e.g. by Aalen et al. (2015) and by Stensrud et al. (2017), naturally fit in this parameterization. However, as mentioned before, the dependence of $T^{a}$ and $T^{0}$ beyond shared frailty is typically not specified. The SCM consists of a joint probability distribution of $(N_{A}, U_{0}, U_{1}, N_{T})$ and a collection of structural assignments $(f_{A}, f_{\lambda })$ such that 
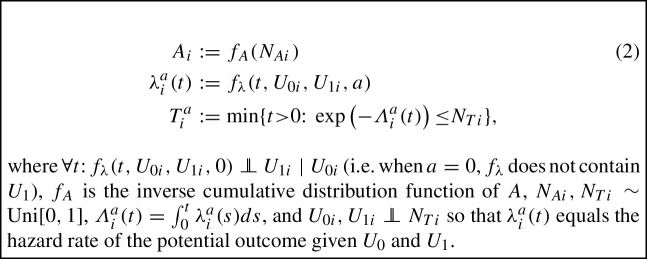


Note that the data generating mechanism is described by this SCM as $T_{i}^{A_{i}} = T_{i}$. If in SCM (2),3then there exists confounding as the distributions of $(U_{0}, U_{1}, N_{T})$ are not exchangeable between exposed and non-exposed individuals. However, in this work we focus on the distribution of data observed from a properly executed RCT, where by the randomization  so that there is no confounding. Note that $\lambda _{i}^{a}(t)$ equals the hazard of the potential outcome of individual *i* under exposure *a*, i.e. 

4 $$\begin{aligned} \lambda _{i}^{a}(t) = \lim _{h\rightarrow 0} h^{-1}{\mathbb {P}}\left( T_{i}^{a} \in [t,t+h) \mid T_{i}^{a} {\ge } t, U_{0i}, U_{1i} \right) , \end{aligned}$$

and is thus a random variable when we consider an arbitrary individual. In this parameterization, $U_{0}$ results in heterogeneity of the hazard under no exposure between individuals, and the presence of $U_{1}$ results in heterogeneity of the effect of the exposure on the hazard between individuals. The SCM could be re-parameterized by including more details, e.g. measured risk factors, so that part of the unmeasured heterogeneity can be explained.

To understand how the so-called built-in selection bias is introduced, realize that $T_{i}^{a}$ depends on *a* and the random variables $U_{0i}$, $U_{1i}$, $N_{Ti}$ only, i.e.  $T_{i}^{a}{:=}~ \min \left\{ t{>}0{:}~ \exp \left( -\int _{0}^{t}f_{\lambda }(s, U_{0i}, U_{1i}, a)ds\right) {\le } N_{Ti}\right\} = g(U_{0i}, U_{1i}, N_{Ti}, a)$, for some function *g*. In an RCT, , so5However, this independence might not hold conditionally on survival at time *t* since $T_{i}{:=}~ g(U_{0i}, U_{1i}, N_{Ti}, A_{i})$, so that $A_{i}{\mid }T_{i}{\ge } t$ can inform on $(U_{0i}, U_{1i}, N_{Ti})$ and thus on $T_{i}^{a}$, then6On the contrary,7as $T_{i}^{a}{:=}~g(U_{0i}, U_{1i},N_{Ti}, a)$, $A_{i}{\mid }T^{a}_{i}{\ge } t$ does not inform on $(U_{0i},U_{1i}, N_{Ti})$. In the literature, the dependence in (6) is often implicitly derived by recognizing that $\{T{\ge } t \}$ is a collider that can thus open a back-door path between *A* and $T^{a}$ (Aalen et al. 2015; Sjölander et al. 2016). The bias that results from conditioning on this collider is referred to as the built-in selection bias of the hazard ratio (Hernán 2010). This complicates causal inference which requires that the distribution of potential outcomes can be expressed in terms of the observed distribution.

In SCM (2), we did not restrict the distribution of $U_{0}$ and $U_{1}$ and only restricted $f_{\lambda }$ and $f_{A}$ to be properly defined hazard and inverse cumulative distribution functions respectively, so that the structural model is very general. It is important to realize that a SCM cannot be validated with data as it describes potential outcomes from different universes. For each individual the outcome can only be observed in one of the universes, and only the fit of the distribution of the outcomes in the factual world can be verified. In this work we focus on settings where the causal effect can be accurately described with a causal hazard ratio, which is defined in the next section. This will be the case when in SCM (2),

$$\begin{aligned} f_{\lambda }(t,U_{0i},U_{1i},a) = f_{0}(t,U_{0i})f_{1}(t,U_{1i},a) \text { and } f_{1}(t,U_{1i},0) = 1. \end{aligned}$$

In the remainder of this manuscript we will restrict ourselves to cause-effect relations that meet this restriction.

## The causal hazard ratio

If $f_{\lambda }(t,U_{0i},U_{1i},a) = f_{0}(t,U_{0i})f_{1}(t,U_{1i},a)$ and $f_{1}(t,U_{1i},0) = 1$, then the individual causal effect is described by $f_{1}(t,U_{1i},a)$. The latter equals the ratio at time *t* of the hazard of an individual’s potential outcome when exposed to level *a* and when not exposed, i.e. $\frac{\lambda _{i}^{a}(t)}{\lambda _{i}^{0}(t)}$. In the case of homogeneous effects, $f_{1}(t, U_{1i}, a) = f_{1}(t, a)$ is equal for all individuals. In the case of heterogeneity of effects, $f_{1}(t, U_{1i}, a)$ is the individual multiplicative causal effect. From a public health perspective, the ratio of the expected hazard rates in the world where everyone is exposed to *a* and in the world where all individuals are not exposed is of interest. This causal hazard ratio (CHR) of interest can be obtained as the ratio of the marginalized (over $U_{0}$ and $U_{1}$) conditional hazard rates in both worlds as presented in Definition 1.

### Definition 1

**Causal hazard ratio** The causal hazard ratio (CHR) for cause-effect relations that can be parameterized with SCM 2 equals

8 $$\begin{aligned} \frac{{\mathbb {E}}\left[ \lambda _{i}^{a}(t)\right] }{{\mathbb {E}}\left[ \lambda _{i}^{0}(t)\right] }&= \frac{\int \lim _{h\rightarrow 0}h^{-1}{\mathbb {P}}\left( T^{a} \in [t,t+h) \mid T^{a}{\ge } t, U_{0}, U_{1} \right) dF_{U_{0}, U_{1}}}{\int \lim _{h\rightarrow 0}h^{-1}{\mathbb {P}}\left( T^{0} \in [t,t+h) \mid T^{0} {\ge } t, U_{0} \right) dF_{U_{0}}}, \end{aligned}$$

where we abbreviate the Lebesque-Stieltjes integral of a function *g* with respect to probability law $F_{X}$, i.e. $\int g(x) dF_{X}(x)$, as $\int g(X) dF_{X}$.

When the parameterization of the cause-effect relations as SCM (2) would be known, the CHR can be expressed in terms of the distribution of the data generating mechanism as presented in Theorem 1.

### Theorem 1

If the cause-effect relations of interest can be parameterized with SCM (2), and  (no confounding), then

$$\begin{aligned} \frac{{\mathbb {E}}\left[ \lambda _{i}^{a}(t)\right] }{{\mathbb {E}}\left[ \lambda _{i}^{0}(t)\right] } = \frac{\int \lim _{h\rightarrow 0}h^{-1}{\mathbb {P}}\left( T \in [t,t+h) \mid T{\ge } t, U_{0}, U_{1},A{=}a \right) dF_{U_{0},U_{1}}}{\int \lim _{h\rightarrow 0}h^{-1}{\mathbb {P}}\left( T \in [t,t+h) \mid T {\ge } t, U_{0}, A{=}0 \right) dF_{U_{0}}}. \end{aligned}$$

For an example, consider the commonly used frailty model where effect heterogeneity is absent, i.e.

$$\begin{aligned} \lambda _{i}^{a}(t) = U_{0i}\lambda _{0}(t)f_{1}(t,a). \end{aligned}$$

The CHR equals the multiplicative effect that does not differ among individuals and equals $f_{1}(t, a)$. By applying Theorem 1, this CHR is indeed derived to equal

$$\begin{aligned} \frac{\int \lim _{h\rightarrow 0} h^{-1}{\mathbb {P}}\left( T \in [t,t+h) \mid T{\ge } t, U_{0}, A{=}a \right) dF_{U_{0}}}{\int \lim _{h\rightarrow 0} h^{-1}{\mathbb {P}}\left( T \in [t,t+h) \mid T{\ge } t, U_{0}, A{=}0 \right) dF_{U_{0}}} = \frac{\lambda _{0}(t){\mathbb {E}}[U_{0}]f_{1}(t,a)}{\lambda _{0}(t){\mathbb {E}}[U_{0}]}. \end{aligned}$$

It is important to note that $f_{1}(t,a)$ deviates from the expected observed hazard ratio equal to

$$\begin{aligned} \frac{\lim _{h\rightarrow 0} h^{-1}{\mathbb {P}}\left( T \in [t,t+h) \mid T{\ge } t, A{=}a \right) }{\lim _{h\rightarrow 0} h^{-1}{\mathbb {P}}\left( T \in [t,t+h) \mid T{\ge } t, A{=}0 \right) } = \frac{\lambda _{0}(t){\mathbb {E}}[U_{0} \mid T{\ge } t, A{=}a]f_{1}(t,a)}{\lambda _{0}(t){\mathbb {E}}[U_{0} \mid T{\ge } t, A{=}0]}, \end{aligned}$$

as we will elaborate on in Sect. 4.

In summary, it became clear that to derive the CHR from data, inference on the distribution of the latent frailty $U_{0}$ and effect modifier $U_{1}$ must be made. When their distributions are known, inference can be drawn from observed data, even when the parameters of the distributions are unknown. Software available to estimate frailty parameters are described by Balan and Putter (2020), and such methods could also be adapted to estimate the latent modifier distribution. However, in practice, the distributions of these latent variables are unknown. Even in the case without causal effect heterogeneity, it is impossible to distinguish the presence of frailty from a time-dependent causal effect (Balan and Putter 2020, Section 2.5). More precisely, different combinations of (varying) effect sizes and frailty distributions give rise to the same marginal distribution. The same applies to combinations that also involve effect modifiers. In the case of clustered survival data (e.g. family data (Valberg et al. 2018)), at least theoretically, the shared frailty could be distinguished from violation of proportional hazards (Balan and Putter 2020). Reasoning along the same lines, individual frailty and marginal time-varying effects could only be derived from effect heterogeneity in the case of recurrent events with stationary distributions.

## Survivor marginalized causal hazard ratio

In Theorem 1, the actual CHR (see Definition 1) has been expressed in terms of the distributions of the observed data, and we concluded that these are not identifiable without making untestable assumptions on the distribution of $(U_{0}, U_{1})$. Instead, practitioners often compute the hazard ratio from data, which expectation we refer to as the observed hazard ratio (OHR) and equals

9 $$\begin{aligned} \text {OHR}(t) = \frac{\lim _{h\rightarrow 0}h^{-1}{\mathbb {P}}\left( T \in [t,t+h) \mid T{\ge } t, A{=}a \right) }{\lim _{h\rightarrow 0}h^{-1}{\mathbb {P}}\left( T \in [t,t+h) \mid T{\ge } t, A{=}0 \right) }. \end{aligned}$$

To be precise, at time *t* the hazard rate can only be observed for non-censored individuals at that time $(C(t)=0)$. However, in this work we will assume independent censoring, so that ${\mathbb {P}}\left( T \mid T{\ge }t, A{=}a \right)$ is equal to ${\mathbb {P}}\left( T \mid T{\ge }t, A{=}a, C(t){=}0\right)$.

To compare the OHR to the CHR that quantifies the causal effect of interest, the OHR should be expressed in terms of potential outcomes. For data from an RCT, by independence (7) and causal consistency, the OHR equals the survivor marginalized causal hazard ratio (SMCHR), i.e. 

10 $$\begin{aligned} \text {SMCHR}(t) = \frac{\lim _{h\rightarrow 0}h^{-1}{\mathbb {P}}\left( T^{a} \in [t,t+h) \mid T^{a}{\ge } t \right) }{\lim _{h\rightarrow 0}h^{-1}{\mathbb {P}}\left( T^{0} \in [t,t+h) \mid T^{0}{\ge } t \right) }. \end{aligned}$$

This SMCHR should not be confused with the ‘marginal causal hazard ratio’ defined by Martinussen et al. (2020) as

11 $$\begin{aligned} \frac{\lim _{h\rightarrow 0}h^{-1}{\mathbb {P}}\left( T^{a} \in [t,t+h) \mid T^{a}{\ge } t, T^{0}{\ge } t \right) }{\lim _{h\rightarrow 0}h^{-1}{\mathbb {P}}\left( T^{0} \in [t,t+h) \mid T^{0}{\ge } t, T^{a}{\ge } t \right) }, \end{aligned}$$

that could also be named the cross-world survivor marginalized causal hazard ratio and is not considered in this work.

We will study how the SMCHR (and thus the OHR from an RCT) differs from the CHR over time. By the law of total probability, the SMCHR in (10) equals

12 $$\begin{aligned} \frac{\lim _{h\rightarrow 0}\int h^{-1}{\mathbb {P}}\left( T^{a} \in [t,t+h) \mid T^{a}{\ge } t, U_{0}, U_{1} \right) dF_{U_{0}, U_{1}{\mid }T^{a}{\ge } t}}{\lim _{h\rightarrow 0}\int h^{-1}{\mathbb {P}}\left( T^{0} \in [t,t+h) \mid T^{0}{\ge } t, U_{0}\right) dF_{U_{0}{\mid }T^{0}{\ge } t}}. \end{aligned}$$

As the integration in the result of Theorem 1 is with respect to the population distribution of $U_{0}$ and $U_{1}$, instead of those individuals for which $T^{a}{\ge } t$ or $T^{0}{\ge } t$, the SMCHR deviates from the CHR, resulting in the built-in selection bias of the hazard (Hernán 2010; Aalen et al. 2015; Stensrud et al. 2018).

The problem induced for estimation of the CHR thus results from inference on a different estimand; the combined effect of the exposure of interest and the difference in latent frailty (and effect modification) distribution. To formalize how (10) deviates from the CHR that equals $\frac{{\mathbb {E}}\left[ f_{0}(t,U_{0})f_{1}(t,U_{1},a)\right] }{{\mathbb {E}}\left[ f_{0}(t,U_{0})\right] }$, we focus on hazard functions that satisfy Condition 1 and do thus not have an infinite discontinuity.

### Condition 1

**Hazard without infinite discontinuity**

$$\begin{aligned} \forall t{>}0{:}~\exists \tilde{{}h}{>}0 \text { such that }\forall h^{*} \in (0,\tilde{{}h}){:}~ {\mathbb {E}}\left[ f_{0}(t+h^{*},U_{0})f_{1}(t+h^{*},U_{1},a) \mid T^{a}{\ge } t\right] {<}\infty \end{aligned}$$

The value of the SMCHR at time *t* is derived in Theorem 2 and can deviate from the CHR.

### Theorem 2

If the cause-effect relations of interest can be parameterized with SCM (2), where

$$\begin{aligned} \lambda _{i}^{a}(t) = f_{0}(t,U_{0i})f_{1}(t,U_{1i},a), \end{aligned}$$

and Condition 1 applies, then

$$\begin{aligned} \frac{\lim _{h\rightarrow 0}h^{-1}{\mathbb {P}}\left( T^{a} \in [t,t+h) \mid T^{a}{\ge } t \right) }{\lim _{h\rightarrow 0}h^{-1}{\mathbb {P}}\left( T^{0} \in [t,t+h) \mid T^{0}{\ge } t \right) } = \frac{{\mathbb {E}}\left[ f_{0}(t,U_{0})f_{1}(t,U_{1},a) \mid T^{a}{\ge } t\right] }{{\mathbb {E}}\left[ f_{0}(t,U_{0}) \mid T^{0}{\ge } t\right] }. \end{aligned}$$

From the proof presented in Appendix A.2, it becomes clear that the conditional expectations that determine the value of the SMCHR equal weighted means of $f_{0}(t,u_{0})f_{1}(t,u_{1},a)$ and $f_{0}(t,u_{0})$ with weights $\tfrac{{\mathbb {P}}(T^a{\ge } t \mid U_{0} = u_{0},U_{1} = u_{1})}{{\mathbb {P}}(T^{a}{\ge } t)}$ and $\tfrac{{\mathbb {P}}(T^0{\ge } t \mid U_{0} = u_{0})}{{\mathbb {P}}(T^{a}{\ge } t)}$ respectively.

To develop our understanding of the difference between the SMCHR and the CHR, we will first continue to study the difference due to frailty and heterogeneity separately in the next two subsections. In the remainder of the section we present examples for cause-effect relations with effect heterogeneity in the presence of frailty, both for independent and dependent $U_{0}$ and $U_{1}$. All programming codes used in the examples presented in this paper can be found online at https://github.com/RAJP93/CHR.

### Causal effect homogeneity

In the case of homogeneous multiplicative causal effects on the hazard, i.e. $f_{1}(t, U_{1i}, a)$ $=$ $f_{1}(t, a)$, the ratio of the marginal hazard rates of individuals satisfying $T_{i}^{a}{\ge } t$ and of those $T_{i}^{0}{\ge } t$ equals $f_{1}(t, a)$ multiplied by a factor that depends on the difference in frailty distributions at time *t* in those two populations as derived in Corollary 1.

#### Corollary 1

If the cause-effect relations of interest can be parameterized with SCM (2), where

$$\begin{aligned} \lambda _{i}^{a}(t) = f_{0}(t,U_{0i})f_{1}(t,a), \end{aligned}$$

and condition 1 applies then

$$\begin{aligned} \frac{\lim _{h\rightarrow 0}h^{-1}{\mathbb {P}}\left( T^{a} \in [t,t+h) \mid T^{a}{\ge } t \right) }{\lim _{h\rightarrow 0}h^{-1}{\mathbb {P}}\left( T^{0} \in [t,t+h) \mid T^{0}{\ge } t \right) } = \frac{{\mathbb {E}}\left[ f_{0}(t,U_{0}) \mid T^{a}{\ge } t\right] }{{\mathbb {E}}\left[ f_{0}(t,U_{0}) \mid T^{0}{\ge } t\right] }f_{1}(t,a). \end{aligned}$$

As stated in the proof in Appendix A.3, the conditional expectation ${\mathbb {E}}\left[ f_{0}(t,U_{0}) \mid T^{a}{\ge } t\right]$ now equals a weighted mean of $f_{0}(t,u_{0})$. The weights equal $\frac{{\mathbb {P}}(T^a{\ge } t \mid U_{0} = u_{0})}{{\mathbb {P}}(T^a{\ge } t)}$ and over time increase for favourable values of $U_{0}$. If $\forall t{>}0{:}~\varLambda ^{a}(u_{0},t)<\varLambda ^{0}(u_{0},t)$, e.g. when $\forall t{>}0{:}~f_{1}(t,a){<}1$, then the weights $\frac{{\mathbb {P}}(T^a{\ge } t \mid U_{0} = u_{0})}{{\mathbb {P}}(T^a{\ge } t)}$ increase slower than $\frac{{\mathbb {P}}(T^0{\ge } t \mid U_{0} = u_{0})}{{\mathbb {P}}(T^0{\ge } t)}$ for favourable values of $u_{0}$, so that for all $t>0{:}$

13 $$\begin{aligned} \frac{{\mathbb {E}}\left[ f_{0}(t,U_{0}) \mid T^{a}{\ge } t\right] }{{\mathbb {E}}\left[ f_{0}(t,U_{0}) \mid T^{0}{\ge } t\right] }>1. \end{aligned}$$

Then, the SMCHR is larger than the CHR at all times. On the contrary, when $\forall t{>}0{:}~\varLambda ^{a}(u_{0},t)>\varLambda ^{0}(u_{0},t)$, then for all $t>0{:}$

14 $$\begin{aligned} \frac{{\mathbb {E}}\left[ f_{0}(t,U_{0}) \mid T^{a}{\ge } t\right] }{{\mathbb {E}}\left[ f_{0}(t,U_{0}) \mid T^{0}{\ge } t\right] }<1, \end{aligned}$$

and the SMCHR is larger than the CHR. An example of the latter was showcased by Stensrud et al. (2017), where a model with $f_{1}(t, a) = 1.81^a$, $f_{0}(u_{0},t) = u_{0}\lambda _{0}(t)$ and compound Poisson distributed frailty $U_{0}$ could well explain the decrease of the effect of hormone replacement therapy on coronary heart disease in postmenopausal women over time as observed from an RCT by the Woman Health Initiative. Based on the same case study, Hernán (2010) explained that even when $f_{1}(t,a)$ is time-invariant the SMCHR is time-varying, as we have formalized in Corollary 1, so that estimates can depend on the follow-up time.

For frailty models as presented by Aalen et al. (2015) and Stensrud et al. (2017), where $f_{0}(t,U_{0i}) = U_{0i}\lambda _{0}(t)$, it has been shown by Balan and Putter (2020) that ${\mathbb {E}}\left[ U_{0} \mid T{\ge } t, A{=}a\right]$ can be expressed in terms of the Laplace transform of the frailty $U_{0}$. Reasoning along the same lines, ${\mathbb {E}}\left[ U_{0} \mid T^{a}{\ge } t\right]$ is expressed in terms of the Laplace transform of the $U_{0}$ in Lemma 1.

#### Lemma 1

If the cause-effect relations of interest can be parameterized with SCM (2), where

$$\begin{aligned} f_{\lambda }(t,U_{0i},U_{1i},a) = U_{0i}\lambda _{0}(t)f_{1}(t,a), \end{aligned}$$

then

15 $$\begin{aligned} {\mathbb {E}}\left[ U_{0} \mid T^{a}{\ge } t\right] =-\frac{{\mathcal {L}}_{U_{0}}^{'}(\int _{0}^{t}\lambda _{0}(s)f_{1}(s,a)ds)}{{{\mathcal {L}}_{U_{0}}}(\int _{0}^{t}\lambda _{0}(s)f_{1}(s,a)ds)}, \end{aligned}$$

where ${{\mathcal {L}}_{U_{0}}}(c) ={\mathbb {E}}\left[ \exp \left( -cU_{0}\right) \right]$ with derivative ${\mathcal {L}}_{U_{0}}^{'}(c)$.

As considered by (Balan and Putter 2020, Figure 5), we present examples with different frailty distributions. To illustrate the so-called selection bias, we consider a binary exposure and let

$$\begin{aligned} \lambda _{i}^{a}(t) = U_{0i}\lambda _{0}(t)\mu ^{a}, \end{aligned}$$

where $\lambda _{0}(t) = \tfrac{t^{2}}{20}$, ${\mathbb {E}}[U_{0}] = 1$ and $\text {var}(U_{0}) = \theta _{0}$ with $U_{0}$ following a Gamma ($\varGamma (\theta _{0}^{-1},\theta _{0})$), inverse Gaussian ($\text {IG}(1,\theta _{0}^{-1})$) or compound Poisson ($\text {CPoi}(3\theta _{0}^{-1},\tfrac{1}{2},\tfrac{2}{3}\theta _{0}$) distribution respectively. The parameterizations, corresponding Laplace transforms and expressions for ${\mathbb {E}}[U_{0} \mid T^{a}{\ge } t]$ can be found in Appendix B. By applying Lemma 1, ${\mathbb {E}}[U_{0} \mid T^{1}{\ge } t]$ and ${\mathbb {E}}[U_{0} \mid T^{0}{\ge } t]$ can be derived. The expressions for these quantities are presented in Table 2 in Appendix C. The SMCHR then follows from Corollary 1 (as the conditional hazard is monotone increasing).

**Fig. 1:**
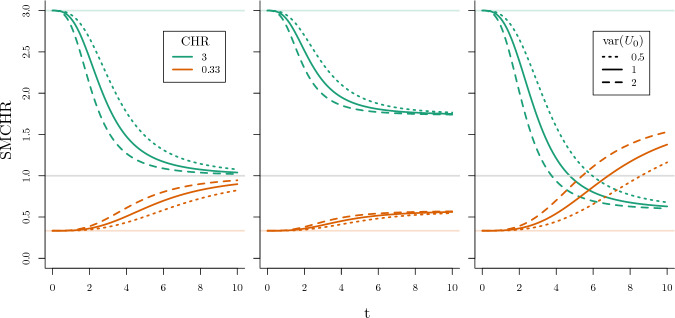
SMCHR over time when $\lambda ^{a}_{i}(t) = U_{0i} \tfrac{t^{2}}{20}\mu ^{a}$ for $\mu = 3$ (green) and $\mu = \tfrac{1}{3}$ (orange), when $U_{0}$ follows a gamma (left), inverse Gaussian (middle) or compound Poisson (right) distribution with variance 0.5 (dotted), 1 (solid) or 2 (dashed) respectively

How the SMCHR deviates from the CHR (equal to $\mu$) over time for $\mu \in \{\tfrac{1}{3}, 3\}$, and $\theta _{0} \in \{0.5, 1, 2\}$ is visualized in Fig. 1.

For both $\mu = \tfrac{1}{3}$ and $\mu = 3$ the selection of individuals that survive time *t* results in a SMCHR that evolves in the opposite direction of the causal effect, towards 1, $\sqrt{\mu }$ and $\sqrt{\mu ^{-1}}$ respectively. For the case of a compound Poisson frailty, the logarithm of this latter limit is even opposite to the sign of the logarithm of the CHR due to the nonsusceptible individuals. For all types of frailty, the higher the variance of $U_{0}$, the larger the difference between the SMCHR and the CHR. For comparison we have also presented the survival curves of $T^{1}$ and $T^{0}$ in Fig. 9 in Appendix E for the setting where $\theta _{0} = 1$. Note that for an RCT, by the independence in (5) and causal consistency, $T^{a}$ follows the same distribution as the time-to-event for individuals exposed to *a* ($T^{a} \overset{d}{=} T {\mid }A{=}a$).

### Causal effect heterogeneity in the absence of frailty

Before we return to the general case presented in Theorem 2, let’s consider the presence of effect heterogeneity in the absence of frailty, i.e.

$$\begin{aligned} f_{\lambda }(t,U_{0i},U_{1i},a) = \lambda _{0}(t)f_{1}(t,U_{1i},a). \end{aligned}$$

If the CHR, ${\mathbb {E}}[f_{1}(t, U_{1i}, a)]$, is equal for all *t*, the SMCHR is not, as over time the exposed individuals that ‘benefit’ more are more likely to survive. The effect of this selection on the SMCHR over time is formalized in Corollary 2.

#### Corollary 2

If the cause-effect relations of interest can be parameterized with SCM (2), where

$$\begin{aligned} \lambda _{i}^{a}(t) = \lambda _{0}(t)f_{1}(t,U_{1i},a), \end{aligned}$$

and Condition 1 applies then

$$\begin{aligned} \frac{\lim _{h\rightarrow 0}h^{-1}{\mathbb {P}}\left( T^{a} \in [t,t+h) \mid T^{a}{\ge } t \right) }{\lim _{h\rightarrow 0}h^{-1}{\mathbb {P}}\left( T^{0} \in [t,t+h) \mid T^{0}{\ge } t \right) }&= {\mathbb {E}}\left[ f_{1}(t,U_{1},a) \mid T^{a}{\ge } t\right] . \end{aligned}$$

The SMCHR thus equals ${\mathbb {E}}\left[ f_{1}(t,U_{1},a) \mid T^{a}{\ge } t\right]$, which is smaller than ${\mathbb {E}}\left[ f_{1}(t,U_{1},a)\right]$ as more weight is placed on lower values of $f_{1}(t,u_{1},a)$ that correspond to higher ${\mathbb {P}}(T^a{\ge } t \mid U_{1} = u_{1})$. Besides the selection of frailty factors, the selection of individual modifiers can thus also lead to selection bias of the estimated hazard ratio. For this hypothetical setting without frailty but with effect heterogeneity, the CHR at *t* is systematically lower (irrespective of whether the exposure is beneficial or harmful on average) than the SMCHR, so the exposure seems more ‘beneficial’ than it is. For a harming exposure, the resulting attenuation of the effect has only been explained due to the presence of frailty and not due to the presence of individual modifiers (Hernán 2010; Stensrud et al. 2017).

Similar to the examples presented in Sect. 4.1, we let $\lambda _{0} = \tfrac{t^{2}}{20}$, ${\mathbb {E}}[U_{1}]=\mu$ and $\text {var}(U_{1})=\theta _{1}$ with $U_{1}$ following a Gamma ($\varGamma (\tfrac{\mu }{\theta _{1}},\tfrac{\theta _{1}}{\mu })$), inverse Gaussian ($\text {IG}(\mu ,\tfrac{\mu ^3}{\theta _{1}})$) or compound Poisson ($\text {CPoi}(3\tfrac{\mu ^2}{\theta _{1}},\tfrac{1}{2},\tfrac{2\theta _{1}}{3\mu })$) distribution respectively. By applying Lemma 1 for $a = 1$ (since $\lambda _{i}^{1}(t) = U_{1i}\lambda _{0}(t)$), we can derive ${\mathbb {E}}[U_{1} \mid T^{1}{\ge } t]$, which by Corollary 2 (as the conditional hazard is monotone increasing) equals the SMCHR, and is presented in Table 3 in Appendix C. Additionally, we derived ${\mathbb {E}}[U_{1} \mid T^{1}{\ge } t]$ for a setting where the multiplicative hazard effect modifier $U_{1}$ equals $\mu _{1}$ (${\le }1$, for individuals that benefit) with probability $p_{1}$, $\mu _{2}$ (${\ge }1$, for individuals that are harmed) with probability $p_{2}$ or 1 (for individuals that are not affected). We define this distribution as the Benefit-Harm-Neutral, $\text {BHN}(p_{1},\mu _{1},p_{2},\mu _{2})$, distribution.

**Fig. 2:**
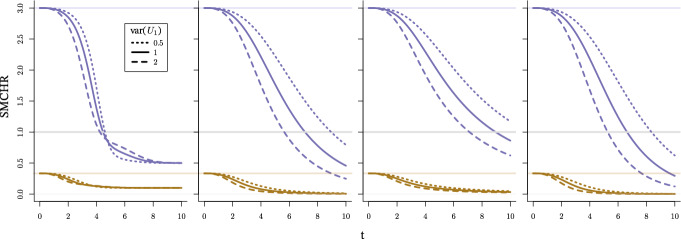
SMCHR over time when $\lambda ^{a}_{i}(t) = \tfrac{t^{2}}{20}(U_{1i})^{a}$ when $U_{1}$ follows a BHN, gamma, inverse Gaussian or compound Poisson distribution (from left to right) with expectation 3 (blue) or $\tfrac{1}{3}$ (brown) and variance 0.5 (dotted), 1 (solid) or 2 (dashed) respectively

For ${\mathbb {E}}[U_{1}] \in \left\{ \tfrac{1}{3}, 3 \right\}$, and $\theta _{1} \in \{0.5, 1, 2\}$ the evolution of the conditional expectation is shown in Fig. 2 for all four effect-modifier distributions. For the $\text {BHN}$ distribution, when ${\mathbb {E}}[U_{1}]$ equals $\tfrac{1}{3}$ and 3, we fix $p_{1} = 0.9$, $\mu _{1} = 0.1$ and $p_{1} = 0.05$, $\mu _{1} = 0.5$ respectively. Expressions for $p_{2}$ and $\mu _{2}$ such that ${\mathbb {E}}[U_{1}] = \mu$ and $\text {var}(U_{1}) = \theta _{1}$ can be found in Appendix B.4.

When the exposure is in expectation harming $({\mathbb {E}}[U_{1}] = 3)$, for all settings considered, there is a point in time that the SMCHR drops below 1. For the continuous distributions, the SMCHR won’t stop decreasing. The decreases for the gamma and compound Poisson settings are very similar, while for the inverse Gaussian setting, this goes a bit slower. For the discrete setting, the SMCHR converges to $\mu _{1}$ of 0.1 and 0.5, respectively. Again, as in the previous subsection, the higher the variability of the latent variable, the faster the SMCHR deviates from the CHR. Only for the discrete effect modifier, the lines cross for the different variances for ${\mathbb {E}}[U_{1} = 3]$, but this is the result of different fractions of individuals that are not affected by the exposure (as the mean and variance are coupled).

### Causal effect heterogeneity in the presence of frailty

In the general case where effect heterogeneity and frailty are present, both heterogeneities affect the value of the SMCHR. By Theorem 2, the ratio evolves as $\frac{{\mathbb {E}}\left[ f_{0}(t,U_{0})f_{1}(t,U_{1},a) \mid T^{a}{\ge } t\right] }{{\mathbb {E}}\left[ f_{0}(t,U_{0}) \mid T^{0}{\ge } t\right] }$. The numerator depends on the joint distribution of $U_{0}$ and $U_{1}$. For illustration, we again consider a binary exposure and let

$$\begin{aligned} f_{\lambda }(t,U_{0i},U_{1i},a) = U_{0i}(U_{1i})^{a}\lambda _{0}(t)f_{1}(t,a), \end{aligned}$$

such that the SMCHR equals $\frac{{\mathbb {E}}\left[ U_{0}U_{1} \mid T^{1}{\ge } t\right] }{{\mathbb {E}}\left[ U_{0} \mid T^{0}{\ge } t\right] }f_{1}(t,1)$ and, by Lemma 1, can be derived from the Laplace transforms of $U_{0}U_{1}$ and $U_{0}$ respectively.

#### Independent $U_{0}$ and $U_{1}$

In the case of independence, the Laplace transform of the product equals ${\mathbb {E}}[{{\mathcal {L}}_{U_{0}}}(c U_{1})]$, which generally does not adopt a tractable form. The case with a discrete effect modifier, introduced in Sect. 4.2, forms an exception. If $U_{1} \sim \text {BHN}(p_{1},\mu _{1},p_{2},\mu _{2}$), then

16 $$\begin{aligned} {\mathcal {L}}_{U_{0}U_{1}}(c) = p_{1}{{\mathcal {L}}_{U_{0}}}(\mu _{1}c)+p_{2}{{\mathcal {L}}_{U_{0}}}(\mu _{2}c)+(1-p_{1}-p_{2}){{\mathcal {L}}_{U_{0}}}(c). \end{aligned}$$

**Fig. 3:**
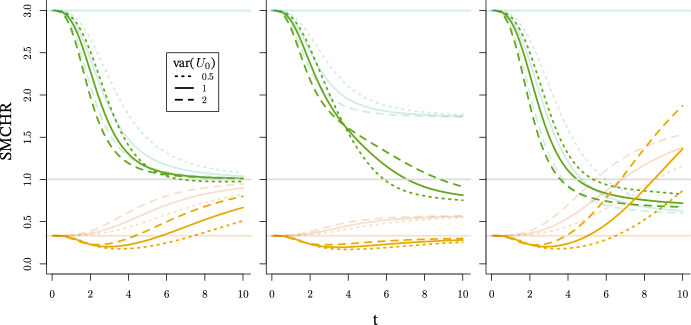
SMCHR over time when $\lambda ^{a}_{i}(t) = U_{0i}(U_{1i})^{a} \tfrac{t^{2}}{20}$ for a unit-variance BHN distributed $U_{1}$ with ${\mathbb {E}}[U_{1}] = 3$ (opaque green) and ${\mathbb {E}}[U_{1}] = \tfrac{1}{3}$ (opaque orange) when $U_{0}$ follows a gamma (left), inverse Gaussian (middle) or compound Poisson (right) distribution with variance 0.5 (dotted), 1 (solid) or 2 (dashed) respectively. For comparison, the lines presented in Fig. 1 are represented by transparent lines

We present the running example where $f_{1}(t,a) = 1$ and $\lambda _{0} = \tfrac{t^{2}}{20}$. As in Sect. 4.1, $U_{0}$ follows a Gamma ($\varGamma (\theta _{0}^{-1},\theta _{0})$), inverse Gaussian ($\text {IG}(1,\theta _{0}^{-1})$) or compound Poisson ($\text {CPois}(3\theta _{0}^{-1},\tfrac{1}{2},\tfrac{2}{3}\theta _{0})$) distribution respectively. Moreover, the latent modifier $U_{1}$ is independent of $U_{0}$ and follows a unit-variance BHN distribution with mean $\mu$. The expressions for ${\mathbb {E}}[U_{0}U_{1} \mid T^{1}{\ge } t]$ are presented in Table 4 in Appendix C. As the ${\mathbb {E}}[U_{0} \mid T^{0}{\ge } t]$ are independent of the $U_{1}$ distribution these expectations are the same as presented in Table 2 in Appedix C. The SMCHR and its limit can be derived by applying Theorem 2 (as the conditional hazard is monotone increasing). Interestingly, for gamma frailty, as in the case without effect heterogeneity, this limit remains 1. For the inverse Gaussian frailty, selection of the effect modifier drastically changes the limit from $\sqrt{{\mathbb {E}}[U_{1}]}$ to $\sqrt{\mu _{1}}$, which is always less or equal to 1. Finally, for compound Poisson frailty the limit changes from $\sqrt{{\mathbb {E}}[U_{1}]^{-1}}$ to $\frac{p_{1}}{\sqrt{\mu _{1}}}+\frac{p_{2}}{\sqrt{\mu _{2}}} + (1-p_{1}-p_{2})$. The evolution of $\frac{{\mathbb {E}} \left[ U_{0}U_{1} \mid T^{1}{\ge } t \right] }{{\mathbb {E}} \left[ U_{0} \mid T^{0}{\ge } t \right] }$ over time is visualized in Fig. 3 for $\theta _{0}\in \{0.5, 1, 2\}$ and $U_{1} \sim \text {BHN}(0.9,0.1,0.03,6.0)$, such that ${\mathbb {E}}[U_{1}] = \tfrac{1}{3}$ and $\text {var}(U_{1}) = 1$, and for $U_{1} \sim \text {BHN}(0.05,0.5,0.82,3.5)$, such that ${\mathbb {E}}[U_{1}] = 3$ and $\text {var}(U_{1}) = 1$.

In the case the CHR is larger than one, the selection of less susceptible individuals (frailty) that are harmed less (effect modifier) in the exposed world, both cause the SMCHR to be smaller than the CHR. Then, the SMCHR decreases faster in the presence of effect heterogeneity. This explains the observation by Stensrud et al. (2017), *“Interestingly, the magnitude of frailty bias is larger when a heterogeneous treatment effect is included"*, for a simulation with frailty and random individual hazard ratios such that ${\mathbb {E}}[\lambda _{i}^{1}(t)] = 1.81>1$. For the gamma and compound Poisson frailty examples, this effect is relatively small as ${\mathbb {E}}[U_{1}U_{0} \mid T^{1}{\ge } t]$ is quite similar to ${\mathbb {E}}[U_{0} \mid T^{1}{\ge } t]$ (presented in Table 4) for the selected $p_{1}, \mu _{1}, p_{2}$ and $\mu _{2}$. However, for inverse Gaussian frailty, the SMCHR deviates much more from the CHR in the presence of effect heterogeneity. In Fig. 7 in Appendix D, the evolution of the SMCHR is presented for a longer timescale, and the limits become apparent.

If the CHR is smaller than one, then the selection of less susceptible individuals (frailty) in the unexposed world and the selection of individuals that benefit more (effect modifier) in the exposed world have opposite effects on the SMCHR. For this case of discrete effect modifiers, the SMCHR first decreases by selecting individuals with more beneficial modifiers and later increases (above the CHR) when the frailty selection effect reveals. For the examples presented, the fraction $p_{1} = 0.9$ of the population with $\mu _{1} = 0.1$ are expected to survive so that over time the SMCHR will resemble the SMCHR in the absence of effect heterogeneity for this subpopulation (with the CHR equal to 0.1). The limit for gamma frailty is still one, so the SMCHR deviates less from the CHR due to the two opposed selection effects. The difference is strongly reduced for the inverse Gaussian frailty as the limit $\sqrt{0.1}$ is close to the actual CHR. Finally, for the compound Poisson frailty, the SMCHR with effect heterogeneity crosses the SMCHR in the absence of effect heterogeneity as the frailty bias is larger for a CHR of 0.1 compared to one of $\tfrac{1}{3}$.

In summary, the bias for the CHR can further increase in the presence of effect heterogeneity, stressing the issues regarding the causal interpretation of OHRs (assuming no confounding). However, for beneficial exposures, the frailty bias can reduce in the presence of effect heterogeneity (e.g. inverse Gaussian frailty), illustrating that there might be settings where the SMCHR is close to the CHR.

#### Dependent $U_{0}$ and $U_{1}$

**Fig. 4:**
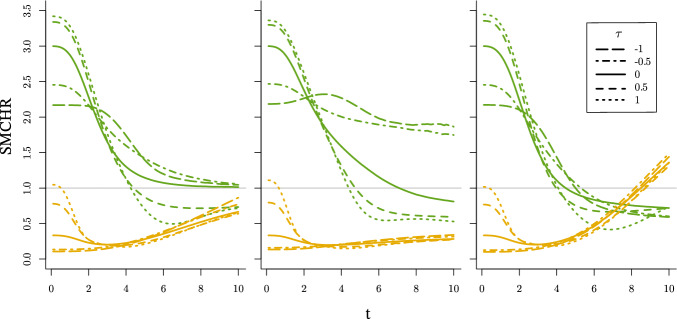
SMCHR over time for $\lambda ^{a}_{i}(t) = U_{0i}(U_{1i})^{a} \tfrac{t^{2}}{20}$, for a unit-variance BHN distributed $U_{1}$ with ${\mathbb {E}}[U_{1}] = 3$ (green) or ${\mathbb {E}}[U_{1}] = \tfrac{1}{3}$ (orange), $U_{0}$ follows a gamma (left), inverse Gaussian (middle) or compound Poisson (right) distribution and the joint distribution of $U_{0}$ and $U_{1}$ follows from a Gaussian copula with varying Kendall’s $\tau$ correlation coefficients (see legend)

In case the multiplicative effect of the exposure on the hazard of susceptible individuals is expected to be higher or lower than for less susceptible individuals, the distribution of $U_{0}U_{1}$ will be less or more variable than when the latent variables are independent. Every bivariate joint distribution function, $F_{(U_{0},U_{1})}$, can be written using the marginal distribution functions and a copula *C* (Sklar 1959). As such,

$$\begin{aligned} F_{(U_{0},U_{1})}(u_{0},u_{1}) = C\left( F_{U_{0}}(u_{0}), F_{U_{1}}(u_{1})\right) \end{aligned}$$

and the Kendall’s $\tau$ correlation coefficient of $U_{0}$ and $U_{1}$ can be written as a function of the copula (Nelsen 2006). To study how the dependence can affect the SMCHR for the setting presented in Fig. 3, we use a Gaussian copula

$$\begin{aligned} C(x,y) = \varPhi _{2,\rho }(\varPhi ^{-1}(x), \varPhi ^{-1}(y)), \end{aligned}$$

where $\varPhi$ and $\varPhi _{2,\rho }$ are the standard normal and bivariate normal with correlation $\rho$ cumulative distribution functions, respectively. For $\rho \in \{-1, \sin (-\tfrac{\pi }{4}), 0, \sin (\tfrac{\pi }{4}), 1\}$ (such that $\tau \in \{-1, -\tfrac{1}{2}, 0, \tfrac{1}{2}, 1\}$) and $\text {var}(U_{0}) = 1$, ${\mathbb {E}}\left[ U_{0}U_{1} \mid T^{1}{\ge } t\right]$ is derived empirically from simulations and are presented in Fig. 8 in Appendix D. The results were very similar when using a Frank, Clayton or Gumbel copula instead of the Gaussian copula. The SMCHRs are presented in Fig. 4.

Note that for $\tau = 0$, we recover the independent setting already shown in Fig. 3 that can be used for comparison. First of all, when $U_{0}$ and $U_{1}$ are dependent, the CHR equals ${\mathbb {E}}[U_{1}]+\text {cov}(U_{0}, U_{1})$. For CHRs greater than one, it becomes clear that the selection effect is more serious for cases with a high positive correlation between $U_{0}$ and $U_{1}$. The stronger selection effect is due to the higher variability of $U_{0}U_{1}$. For CHRs less than one, this trend is only true at short timescales, after which the frailty selection effect takes over since, for this example, for a large fraction of the individuals, $p_{1} = 0.9$, the effect is the same ($U_{1} = 0.1$).

**Fig. 5:**
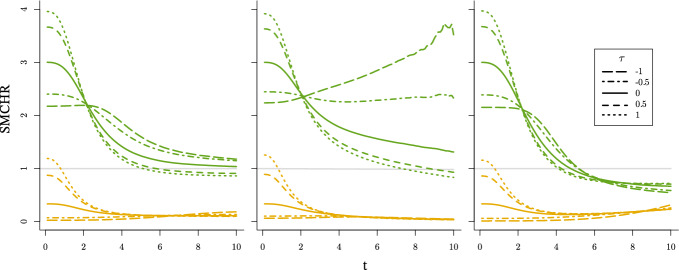
SMCHR over time for $\lambda ^{a}_{i}(t) = U_{0i}(U_{1i})^{a} \tfrac{t^{2}}{20}$, for a unit-variance gamma distributed $U_{1}$ with ${\mathbb {E}}[U_{1}] = 3$ (green) or ${\mathbb {E}}[U_{1}] = \tfrac{1}{3}$ (orange), $U_{0}$ follows a gamma (left), inverse Gaussian (middle) or compound Poisson (right) distribution and the joint distribution of $U_{0}$ and $U_{1}$ follows from a Gaussian copula with varying Kendall’s $\tau$ correlation coefficients (see legend)

When we use a continuous gamma distributed $U_{1}$ instead, the frailty selection effect is less apparent, as shown in Fig. 5.

So far, for CHRs larger than one, we have observed a monotonic SMCHR. However, in the case of strong dependence between $U_{0}$ and $U_{1}$ ($|\tau | = 1$), for inverse Gaussian frailty, ${\mathbb {E}}[U_{0} \mid T^{0}{\ge } t]$ decreases faster than ${\mathbb {E}}[U_{0}U_{1} \mid T^{1}{\ge } t]$ resulting in a non-monotonic trend for the SMCHR. For a Gamma distributed $U_{1}$, in the case of inverse Gaussian distributed $U_{0}$ with $\tau = 1$, the SMCHR even equals a monotonic increasing function over time as shown in Fig. 5.

In Sect. 4, we have derived and applied Theorem 1 to several examples to illustrate the deviation of the SMCHR from the CHR. In summary, even when the CHR is constant, an OHR from an RCT equal to a particular value *x* (at time *t*) can occur for different CHR values when the $(U_{0}, U_{1})$ distribution is unknown as summarized in Table 1.

**Table 1:** Assuming no confounding, an OHR (at time *t*) equal to a particular value *x* can occur for all values of a constant CHR as a result of selection of the frailty $(U_{0})$ or modifier $(U_{1})$ which might be dependent

		Cause	Presented examples
$x{>}1$	$\text {CHR}{>}x$	Frailty or modifier selection	Figs. 1, 2 and 3
	$1{<}\text {CHR}{<}x$	Dependence $U_{0}$ and $U_{1}$	Inverse Gaussian frailty - $\tau = 1$ (Figs. 4 and 5)
	$\text {CHR}{<}1$	Frailty selection	Compound Poisson frailty (Figs. 1 and 3)
$x{<}1$	$\text {CHR}{>}1$	Frailty or modifier selection	Compound Poisson frailty (Figs. 1 and 3)
	$x{<}\text {CHR}{<}1$	Modifier selection	Gamma distributed modifier (Fig. 5)
	$\text {CHR}{<}x$	Frailty selection	Figs. 1 and 3

## Implications for the Cox model

**Fig. 6:**
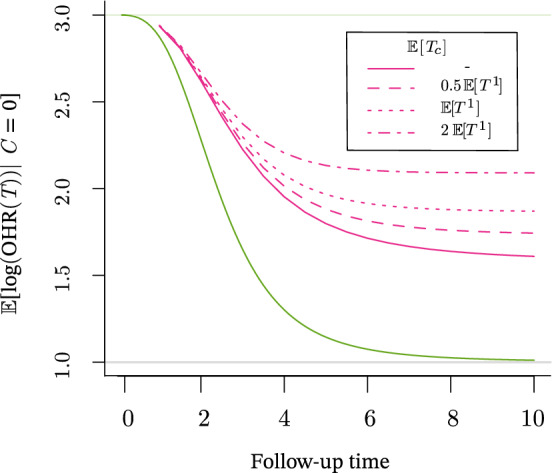
Empirically obtained $\exp \left( {\mathbb {E}}[\log (\text {OHR}(T)) \mid C = 0]\right)$ for an increasing time to follow-up (solid pink), and cases with an additional exponentially distributed censoring time ($T_{C}$, dashed pink), when $\lambda ^{a}_{i}(t) = U_{0i}(U_{1i})^{a} \tfrac{t^{2}}{20}$, $U_{1} \sim \text {BHN}(0.05,0.5,0.82,3.5)$ and $U_{0} \sim \varGamma (1,1)$. The SMCHR is also presented (green)

**Fig. 7:**
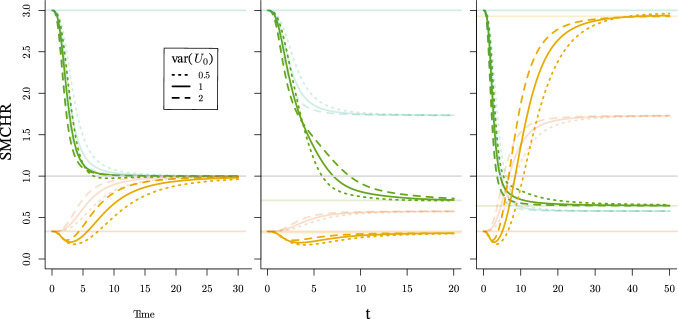
SMCHR over time when $\lambda ^{a}_{i}(t) = U_{0i}(U_{1i})^{a} \tfrac{t^{2}}{20}$ for a unit-variance BHN distributed $U_{1}$ with ${\mathbb {E}}[U_{1}] = 3$ (opaque green) and ${\mathbb {E}}[U_{1}] = \tfrac{1}{3}$ (opaque orange) when $U_{0}$ follows a gamma (left), inverse Gaussian (middle) or compound Poisson (right) distribution with variance 0.5 (dotted), 1 (solid) or 2 (dashed) respectively. For comparison, the lines presented in Fig. 1 as well as the limits of the SMCHRs are represented by transparent lines

**Fig. 8:**
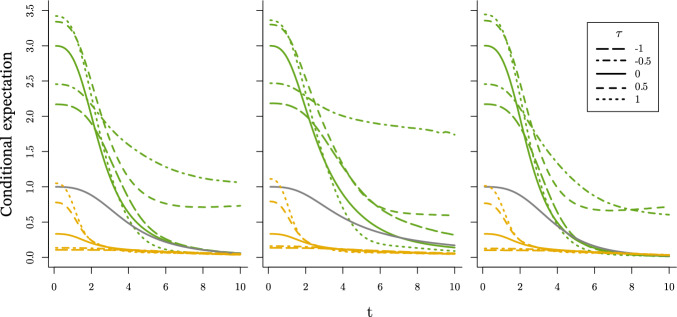
${\mathbb {E}}[U_{0}U_{1} \mid T^{1}{\ge } t]$ over time for $\lambda ^{a}_{i}(t) = U_{0i}(U_{1i})^{a} \tfrac{t^{2}}{20}$, for a unit-variance BHN distributed $U_{1}$ with ${\mathbb {E}}[U_{1}] = 3$ (green) or ${\mathbb {E}}[U_{1}] = \tfrac{1}{3}$ (orange), $U_{0}$ follows a gamma (left), inverse Gaussian (middle) or compound Poisson (right) distribution and the joint distribution of $U_{0}$ and $U_{1}$ follows from a Gaussian copula with varying Kendall’s $\tau$ correlation coefficients (see legend). Furthermore, the evolution of ${\mathbb {E}}[U_{0} \mid T^{0}{\ge } t]$ is presented (grey)

**Fig. 9:**
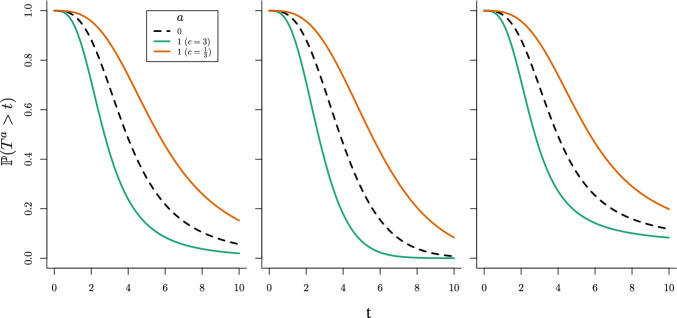
Survival curves for $T^{0}$ (black), and $T^{1}$ (coloured) when $\lambda ^{a}_{i}(t) = U_{0i} \tfrac{t^{2}}{20}\mu ^{a}$ for $\mu = 3$ (green) and $\mu = \tfrac{1}{3}$ (orange), when $U_{0}$ follows a gamma, inverse Gaussian or compound Poisson distribution (from left to right) with variance 1. The corresponding SMCHR curves were presented in Fig. 1

We have demonstrated that in the presence of frailty and effect heterogeneity, even when the CHR is time-invariant, the SMCHR varies over time. Then, the proportional hazards assumption will not hold for an observed hazard ratio from an RCT (that is, with independent censoring, equal to the SMCHR as discussed at the start of Sect. 4). Despite the many options to deal with non-proportional hazards (see, e.g. (Thernau and Grambsch 2000, Section 6.5) or Bennett 1983; Hess 1994; Wei and Schaubel 2008), in the majority of epidemiological time-to-event studies, the misspecified traditional Cox’s proportional hazard model is fitted. The logarithm of the Cox estimate can be interpreted as the logarithm of the OHR marginalized over the observed death times (Schemper et al. 2009), i.e. ${\mathbb {E}}[\log (\text {OHR}(T)) \mid C = 0]$ for censoring indicator *C*. The logarithm of the Cox estimate obtained from an RCT thus equals a time-weighted average of the logarithm of the OHR. In the case of non-proportional hazards, even for independent censoring, the estimate is well-known to be affected by the censoring distribution. It differs from the average log hazard ratio ${\mathbb {E}}\left[ \log (\text {OHR}(T))\right]$ (Xu and O’Quigley 2000; Schemper et al. 2009; Boyd et al. 2012). Therefore, the bias of the Cox estimate, when the estimand is the CHR, will depend on the joint distribution of $(U_0, U_1)$ as well as the censoring distribution. In most cases considered in Sect. 4, the deviation of the OHR from the CHR increased over time. For independent censoring, the probability of censoring increases over time, so the Cox estimate is closer to the OHR at short times. In Fig. 6, this is demonstrated for the gamma-frailty case ($\text {var}(U_{0}) = 1$, for which the SMCHR was presented in Fig. 3) by presenting empirically obtained ${\mathbb {E}}[\log (\text {OHR}(T)) \mid C = 0]$ (with 1, 000, 000 replications) based on a varying follow-up time and loss to follow-up modelled with an exponential censoring-time distribution with varying means.

A time-varying OHR violates the proportional hazard assumption that can be verified when fitting a Cox model. When the assumption is not rejected in practice, the statistical test used is probably underpowered. In the presence of heterogeneity, only when the actual CHR would be time-varying, the OHR can be approximately constant when the selection effect and the change in CHR roughly cancel out (Stensrud et al. 2018; Stensrud and Hernán 2020). The data cannot be used to distinguish the latter case from the case with a constant CHR but no heterogeneity and, thus, no selection effect. Similarly, as mentioned at the end of Sect. 3, when the OHR would vary over time, we can never conclude whether this is the result of a time-varying causal effect or due to selection. However, the proportional hazard assumption would be violated in both cases, and a standard Cox model is inappropriate.

## Discussion

In this paper, we have formalized how heterogeneity leads to deviation of the SMCHR (see Equation (10)) from the CHR of interest (see Definition 1) due to the selection of both the individual frailty factor $(U_{0})$ and the individual effect modifier $(U_{1})$. This work generalizes frailty examples presented in the literature (Hernán 2010; Aalen et al. 2015; Stensrud et al. 2017), by considering the possibility of multiplicative effect (on the hazard) heterogeneity that also results in non-exchangeability of exposed and unexposed individuals over time. As a result of the individual effect modifier ($U_{1}$), the individuals that survive in the exposed groups are expected to benefit more or suffer less from the exposure. At the same time $U_{0}{\mid }T^{1}{\ge } t$ will have a different distribution than $U_{0}{\mid }T^{0}{\ge } t$. When the CHR is larger than one, and , the selection effects act in the same direction. On the other hand, when the CHR is smaller than one and , the selection effects can act in opposite directions so that the SMCHR might be closer to the CHR than in the case without effect heterogeneity (see Fig. 3).

For data from an RCT, with independent censoring, the expected observed hazard ratio equals the studied SMCHR so that all results directly relate to this OHR. For observational data, the OHR does not equal the SMCHR due to confounding. However, when all confounders ${\varvec{L}}$ are observed, i.e. , one can study the conditional (on ${\varvec{L}}$) OHR that in turn is equal to the conditional SMCHR. The presented theorems are valid while conditioning on ${\varvec{L}}$.

The intuition explained by Hernán (2010) suggests that an appropriate estimate of the SMCHR is expected to underestimate the actual effect size, while the sign of the logarithms of the SMCHR and the CHR are equal. However, we have shown that in the presence of effect heterogeneity, an SMCHR equal to a particular value *x* can occur both under $\text {CHR}{>}1$ as well as $\text {CHR}{<}1$ as summarized in Table 1. Therefore, OHRs from RCTs are not guaranteed to present a lower bound for the causal effect without making untestable assumptions on the $(U_{0}, U_{1})$ distribution. We have derived how the SMCHR will evolve due to the selection of frailty and effect modifiers in Theorem 1. However, in practice, only the evolution of the OHR can be found (assuming sufficient data is available). Even after assuming the absence of confounding (e.g. for an RCT), the CHR is non-identifiable without making (untestable) assumptions on the $(U_{0}, U_{1})$ distribution as discussed at the end of Sect. 3. We can thus not distinguish between a time-varying CHR without selection of $U_{0}$ and $U_{1}$ or a time-invariant CHR with selection, see e.g. Stensrud and Hernán (2020). Adjusting for other risk factors can lower the remaining variability of $U_{0}$ and $U_{1}$ so that the difference between the conditional OHR and CHR is reduced. Even for an RCT, it may thus help to focus on adjusted hazard ratios despite the absence of confounding. Nevertheless, adjusting for other risk factors will require more data and modelling decisions.

Finally, we want to remark that for cause-effect relations that cannot be described by SCM (2) with $f_{\lambda }(t,U_{0i},U_{1i},a) = f_{0}(t,U_{0i})f_{1}(t,U_{1i},a)$, the CHR is not the appropriate measure to quantify the causal effect. Then, other causal hazard contrasts can be relevant that may or may not have an observable analog. For example, additive hazard models (when well-specified) do not suffer from the frailty selection as shown by Aalen et al. (2015), but these models will still suffer from latent modifier selection in the presence of effect heterogeneity (${\mathbb {E}}[U_{1} \mid T^{1}{\ge } t]{>}{\mathbb {E}}[U_{1}]$) as demonstrated in our companion paper (Post et al. 2024).

We hope that the discussed effect heterogeneity and formalization of the built-in selection bias of the OHR show the need to use more suitable estimands. As suggested by others, contrasts of the survival probabilities, the median, or the restricted mean survival time of potential outcomes are proper measures to quantify causal effects on time-to-event outcomes (Hernán 2010; Stensrud et al. 2018; Bartlett et al. 2020; Young et al. 2020). Modelling and estimating hazard rates can still be helpful for causal inference when the hazards are used to derive one of the appropriate causal estimands (Ryalen et al. 2018).

## Figures and Tables

**Fig. 10:**
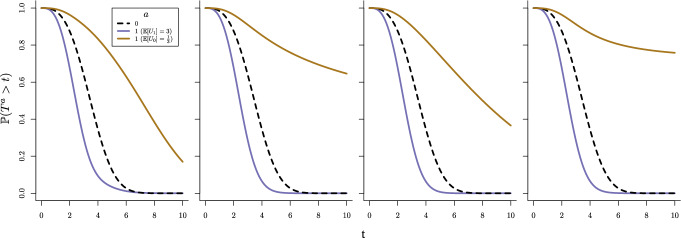
Survival curves for $T^{0}$ (black), and $T^{1}$ (coloured) when $\lambda _{i}^{a}(t) = U_{0i} \tfrac{t^{2}}{20}(U_{1i})^{a}$, when $U_{1}$ follows a BHN, gamma, inverse Gaussian or compound Poisson distribution (from left to right) with expectation 3 (blue) or $\tfrac{1}{3}$ (brown) and variance 1. The corresponding SMCHR curves were presented in Fig. 2

**Fig. 11:**
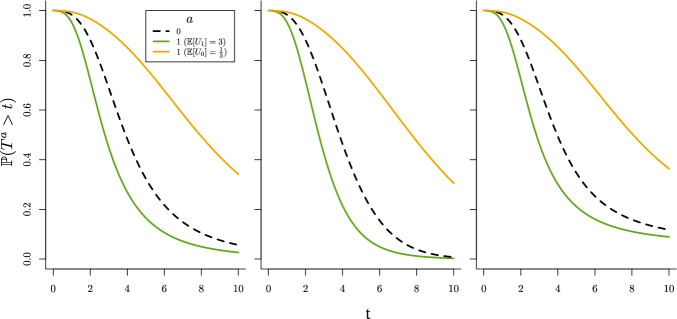
Survival curves for $T^{0}$ (black), and $T^{1}$ (coloured) when $\lambda _{i}^{a}(t) = U_{0i}(U_{1i})^{a} \tfrac{t^{2}}{20}$ for a unit-variance BHN distributed $U_{1}$ with ${\mathbb {E}}[U_{1}] = 3$ (green) and ${\mathbb {E}}[U_{1}] = \tfrac{1}{3}$ (orange) when $U_{0}$ follows a gamma, inverse Gaussian or compound Poisson distribution (from left to right) with variance 1. The corresponding SMCHR curves were presented in Fig. 3

**Fig. 12:**
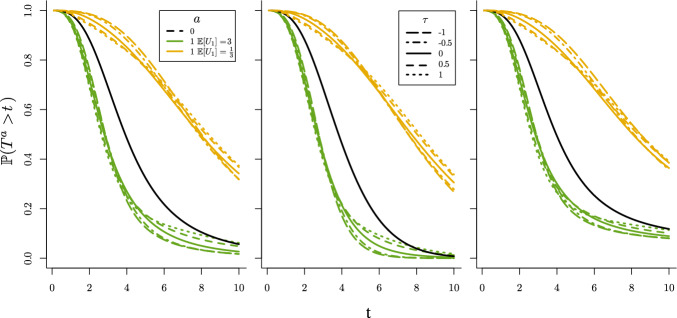
Survival curves for $T^{0}$ (black), and $T^{1}$ (coloured) when $\lambda ^{a}_{i}(t) = U_{0i}(U_{1i})^{a} \tfrac{t^{2}}{20}$, for a unit-variance BHN distributed $U_{1}$ with ${\mathbb {E}}[U_{1}] = 3$ (green) or ${\mathbb {E}}[U_{1}] = \tfrac{1}{3}$ (orange), when $U_{0}$ follows a gamma, inverse Gaussian or compound Poisson distribution (from left to right) with variance 1 and the joint distribution of $U_{0}$ and $U_{1}$ follows from a Gaussian copula with varying Kendall’s $\tau$. The corresponding SMCHR curves were presented in Fig. 4

**Fig. 13:**
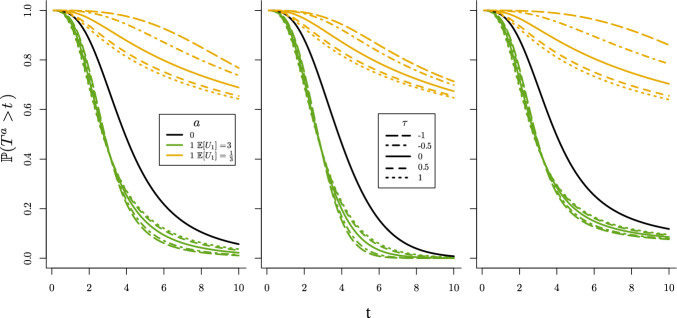
Survival curves for $T^{0}$ (black), and $T^{1}$ (coloured) when $\lambda _{i}^{a}(t) = U_{0i}(U_{1i})^{a} \tfrac{t^{2}}{20}$, for a unit-variance gamma distributed $U_{1}$ with ${\mathbb {E}}[U_{1}] = 3$ (green) or ${\mathbb {E}}[U_{1}] = \tfrac{1}{3}$ (orange) when $U_{0}$ follows a gamma, inverse Gaussian or compound Poisson distribution (from left to right) with variance 1. and the joint distribution of $U_{0}$ and $U_{1}$ follows from a Gaussian copula with varying Kendall’s $\tau$. The corresponding SMCHR curves were presented in Fig. 5

## Proofs

### Proof of theorem 1

#### Proof

$$\begin{aligned} \frac{{\mathbb {E}}\left[ \lambda _{i}^{a}(t)\right] }{{\mathbb {E}}\left[ \lambda _{i}^{0}(t)\right] }&= \frac{{\mathbb {E}}\left[ f_{0}(t,U_{0i})f_{1}(a,U_{1i},t)\right] }{{\mathbb {E}}\left[ f_{0}(t,U_{0i})\right] }\\&= \frac{\int \lim _{h\rightarrow 0}h^{-1}{\mathbb {P}}\left( T^{a} \in [t,t+h) \mid T^{a} {\ge } t, U_{0}, U_{1} \right) dF_{U_{0}, U_{1}}}{\int \lim _{h\rightarrow 0}h^{-1}{\mathbb {P}}\left( T^{0} \in [t,t+h) \mid T^{0}{\ge } t, U_{0} \right) dF_{U_{0}}}, \end{aligned}$$

By randomization the independence in (7) applies and the CHR is equal to

$$\begin{aligned} \frac{\int \lim _{h\rightarrow 0}h^{-1}{\mathbb {P}}\left( T^{a} \in [t,t+h) \mid T^{a} {\ge } t, U_{0}, U_{1},A{=}a \right) dF_{U_{0}, U_{1}}}{\int \lim _{h\rightarrow 0}h^{-1}{\mathbb {P}}\left( T^{0} \in [t,t+h) \mid T^{0}{\ge } t, U_{0}, A{=}0 \right) dF_{U_{0}}}. \end{aligned}$$

Finally, by causal consistency,

$$\begin{aligned} \frac{{\mathbb {E}}\left[ \lambda _{i}^{a}(t)\right] }{{\mathbb {E}}\left[ \lambda _{i}^{0}(t)\right] }=\frac{\int \lim _{h\rightarrow 0}h^{-1}{\mathbb {P}}\left( T \in [t,t+h) \mid T{\ge } t, U_{0}, U_{1},A{=}a \right) dF_{U_{0}, U_{1}}}{\int \lim _{h\rightarrow 0}h^{-1}{\mathbb {P}}\left( T \in [t,t+h) \mid T{\ge } t, U_{0}, A{=}0 \right) dF_{U_{0}}}. \square \end{aligned}$$

### Proof of theorem 2

#### Proof

By the law of total probability,

$$\begin{aligned}&\frac{\lim _{h\rightarrow 0}h^{-1}{\mathbb {P}}\left( T^{a} \in [t,t+h) \mid T^{a}{\ge } t \right) }{\lim _{h\rightarrow 0}h^{-1}{\mathbb {P}}\left( T^{0} \in [t,t+h) \mid T^{0}{\ge } t \right) } = \\&\frac{\lim _{h\rightarrow 0}\int h^{-1}{\mathbb {P}}\left( T^{a} \in [t,t+h) \mid T^{a}{\ge } t, U_{0}, U_{1} \right) dF_{U_{0},U_{1}{\mid }T^{a}{\ge } t}}{\lim _{h\rightarrow 0}\int h^{-1}{\mathbb {P}}\left( T^{0} \in [t,t+h) \mid T^{0}{\ge } t, U_{0} \right) dF_{U_{0}{\mid }T^{0}{\ge } t}} \end{aligned}$$

First we focus on the integrand,

$$\begin{aligned}&h^{-1}{\mathbb {P}}\left( T^{a} \in [t,t+h) \mid T^{a}{\ge } t, U_{0}, U_{1} \right) \\ {}&= h^{-1}\frac{{\mathbb {P}}\left( T^{a} {\ge } t \mid U_{0}, U_{1} \right) - {\mathbb {P}}\left( T^{a} {\ge } t + h \mid U_{0}, U_{1} \right) }{{\mathbb {P}}\left( T^{a} {\ge } t \mid U_{0}, U_{1} \right) } \\&= h^{-1}\left( 1-\frac{{\mathbb {P}}\left( T^{a} {\ge } t + h \mid U_{0}, U_{1} \right) }{{\mathbb {P}}\left( T^{a} {\ge } t \mid U_{0}, U_{1} \right) }\right) \\&= h^{-1}\left( 1-\frac{\exp \left( - \int _{0}^{t+h} f_{0}(s,U_{0})f_{1}(s,U_{1},a) ds \right) }{\exp \left( - \int _{0}^{t} f_{0}(s,U_{0})f_{1}(s,U_{1},a) ds \right) }\right) \\&= h^{-1}\left( 1-\exp \left( - \int _{t}^{t+h} f_{0}(s,U_{0})f_{1}(s,U_{1},a) ds \right) \right) \end{aligned}$$

For monotonic (increasing or decreasing) conditional hazard functions if $h_{2}{<}h_{1}$, then

$$\begin{aligned}{} & {} h_{1}^{-1}\left( 1- \exp \left( - \int _{t}^{t+h_{1}} f_{0}(s,U_{0})f_{1}(s,U_{1},a) ds \right) \right) \\{} & {} \quad {\le } h_{2}^{-1}\left( 1- \exp \left( - \int _{t}^{t+h_{2}} f_{0}(s,U_{0})f_{1}(s,U_{1},a) ds \right) \right) \end{aligned}$$

or

$$\begin{aligned}{} & {} h_{1}^{-1}\left( 1- \exp \left( - \int _{t}^{t+h_{1}} f_{0}(s,U_{0})f_{1}(s,U_{1},a) ds \right) \right) \\{} & {} \quad {\ge } h_{2}^{-1}\left( 1- \exp \left( - \int _{t}^{t+h_{2}} f_{0}(s,U_{0})f_{1}(s,U_{1},a) ds \right) \right) \end{aligned}$$

as the average integrated conditional hazard over the interval increases (or decreases). Moreover,

$$\begin{aligned} \lim _{h\rightarrow 0} h^{-1}{\mathbb {P}}\left( T^{a} \in [t,t+h) \mid T^{a}{\ge } t, U_{0}, U_{1} \right) =f_{0}(s,U_{0})f_{1}(s,U_{1},a)\ge 0. \end{aligned}$$

Then, the limit and integral can be interchanged by directly applying the monotone convergence theorem.

For non-monotone conditional hazard functions, when Condition 1 applies, for every *t*, there exist a ${\tilde{h}}$ so that $\forall h^{*} \in (0,\tilde{{}h}){:}~ {\mathbb {E}}\left[ f_{0}(t+h^{*},U_{0})f_{1}(t+h^{*},U_{1},a) \mid T^{a}{\ge } t\right] {<}\infty$. Moreover, let $t^{*}{=}~ \mathop {\mathrm {arg\,max}}\limits _{s \in (t, t + \tilde{{}h})} f_{0}(s,U_{0})f_{1}(s,U_{1},a),$ so that for $h<{\tilde{h}}{:}~$

$$\begin{aligned} h^{-1}{\mathbb {P}}\left( T^{a} \in [t,t+h) \mid T^{a}{\ge } t, U_{0}, U_{1} \right) \le h^{-1}\left( 1- \exp \left( - h f_{0}(t^{*},U_{0})f_{1}(t^{*},U_{1},a)\right) \right) . \end{aligned}$$

Using the power series definition of the exponential function,

$$\begin{aligned}&h^{-1}{\mathbb {P}}\left( T^{a} \in [t,t+h) \mid T^{a}{\ge } t, U_{0}, U_{1} \right) \\&\quad {\le } h^{-1}\left( 1- \frac{1}{\sum _{k = 0}^{\infty }h^{k} (f_{0}(t^{*},U_{0})f_{1}(t^{*},U_{1},a))^{k}\tfrac{1}{k!}}\right) \\&\quad = h^{-1} \frac{\sum _{k = 1}^{\infty }h^{k} (f_{0}(t^{*},U_{0})f_{1}(t^{*},U_{1},a))^{k}\tfrac{1}{k!}}{\sum _{k = 0}^{\infty }h^{k} (f_{0}(t^{*},U_{0})f_{1}(t^{*},U_{1},a))^{k}\tfrac{1}{k!}}\\&\quad = f_{0}(t^{*},U_{0})f_{1}(t^{*},U_{1},a) \frac{\sum _{k = 1}^{\infty }h^{k-1} (f_{0}(t^{*},U_{0})f_{1}(t^{*},U_{1},a))^{k-1}\tfrac{1}{k!}}{\sum _{k = 0}^{\infty }h^{k} (f_{0}(t^{*},U_{0})f_{1}(t^{*},U_{1},a))^{k}\tfrac{1}{k!}}\\&\quad = f_{0}(t^{*},U_{0})f_{1}(t^{*},U_{1},a) \frac{\sum _{k = 0}^{\infty }h^{k} (f_{0}(t^{*},U_{0})f_{1}(t^{*},U_{1},a))^{k}\tfrac{1}{(k+1)!}}{\sum _{k = 0}^{\infty }h^{k} (f_{0}(t^{*},U_{0})f_{1}(t^{*},U_{1},a))^{k}\tfrac{1}{k!}}\\&\quad {<}~f_{0}(t^{*},U_{0})f_{1}(t^{*},U_{1},a). \end{aligned}$$

Furthermore, ${\mathbb {E}}\left[ f_{0}(t^{*},U_{0})f_{1}(t^{*},U_{1},a) \mid T^{a}{\ge } t\right] {<}\infty$ when $\forall h {\in }(0,\tilde{{}h}) {:}~ {\mathbb {E}}[f_{0}(t+h,U_{0})$ $f_{1}(t+h,U_{1},a) \mid T^{a}{\ge } t] < \infty$. Then we can change the order of the limit and integral by application of the dominated convergence theorem and conclude,

$$\begin{aligned}{} & {} \frac{\lim _{h\rightarrow 0}\int h^{-1}{\mathbb {P}}\left( T^{a} \in [t,t+h) \mid T^{a}{\ge } t, U_{0}, U_{1} \right) dF_{U_{0},U_{1}{\mid }T^{a}{\ge } t}}{\lim _{h\rightarrow 0}\int h^{-1}{\mathbb {P}}\left( T^{0} \in [t,t+h) \mid T^{0}{\ge } t, U_{0} \right) dF_{U_{0}{\mid }T^{0}{\ge } t}}\\{} & {} \quad = \frac{{\mathbb {E}}\left[ f_{0}(t,U_{0})f_{1}(t,U_{1},a) \mid T^{a}{\ge } t\right] }{{\mathbb {E}}\left[ f_{0}(t,U_{0}) \mid T^{0}{\ge } t\right] }. \end{aligned}$$

Applying Bayes rule, we obtain

$$\begin{aligned}&{\mathbb {E}}\left[ f_{0}(t,U_{0})f_{1}(t,U_{1},a) \mid T^{a}{\ge } t\right] \\&\quad = \int f_{0}(t,U_{0})f_{1}(t,U_{1},a) dF_{(U_{0},U_{1}) \mid T^{a}{\ge } t}\\&\quad = \int f_{0}(t,U_{0})f_{1}(t,U_{1},a)\frac{ {\mathbb {P}}(T^a{\ge } t \mid U_{0},U_{1})}{{\mathbb {P}}(T^{a}{\ge } t)}dF_{(U_{0},U_{1})}\\&\quad = \int f_{0}(t,U_{0})f_{1}(t,U_{1},a)\frac{\exp \left( -\int _{0}^{t}f_{0}(s,U_{0})f_{1}(s,U_{1},a)ds\right) }{\int \exp \left( -\int _{0}^{t}f_{0}(s,U_{0})f_{1}(s,U_{1},a)ds\right) dF_{(U_{0},U_{1})}}dF_{(U_{0},U_{1})}. \end{aligned}$$

Furthermore,

$$\begin{aligned}&{\mathbb {E}}\left[ f_{0}(t,U_{0}) \mid T^{a}{\ge } t\right] \\&\quad = \int f_{0}(t,U_{0}) dF_{U_{0}{\mid }T^{a}{\ge } t}\\&\quad = \int f_{0}(t,U_{0})\frac{ {\mathbb {P}}(T^a{\ge } t \mid U_{0})}{{\mathbb {P}}(T^{a}{\ge } t)}dF_{U_{0}}\\&\quad = \int f_{0}(t,U_{0})\frac{\int \exp \left( -\int _{0}^{t}f_{0} (s,U_{0})f_{1}(s,U_{1},a)ds\right) dF_{U_{1} \mid U_{0}}}{ \int \exp \left( -\int _{0}^{t}f_{0}(s,U_{0})f_{1}(s,U_{1},a)ds\right) dF_{(U_{0},U_{1})}}dF_{U_{0}}, \end{aligned}$$

such that for $a = 0$,

$$\begin{aligned} {\mathbb {E}}\left[ f_{0}(t,U_{0}) \mid T^{0}{\ge } t\right] = \int f_{0}(t,U_{0}) \frac{\exp \left( -\int _{0}^{t}f_{0}(s,U_{0})ds\right) }{\int \exp \left( -\int _{0}^{t}f_{0}(s,U_{0})ds\right) dF_{U_{0}}}dF_{U_{0}}. \end{aligned}$$

The ratio of both gives us the result. $\square$

### Proof of corollary 1

#### Proof

By Theorem 2,

$$\begin{aligned} \frac{\lim _{h\rightarrow 0}h^{-1}{\mathbb {P}}\left( T^{a} \in [t,t+h) \mid T^{a}{\ge } t \right) }{\lim _{h\rightarrow 0}h^{-1}{\mathbb {P}}\left( T^{0} \in [t,t+h) \mid T^{0}{\ge } t \right) } = \frac{{\mathbb {E}}\left[ f_{0}(t,U_{0})f_{1}(t,U_{1},a) \mid T^{a}{\ge } t\right] }{{\mathbb {E}}\left[ f_{0}(t,U_{0}) \mid T^{0}{\ge } t\right] }. \end{aligned}$$

and equals

$$\begin{aligned}{} & {} \int f_{0}(t,U_{0})f_{1}(t,U_{1},a)\tfrac{\exp \left( -\varLambda ^{a}(t,U_{0},U_{1})\right) }{\int \exp \left( -\varLambda ^{a}(t,U_{0},U_{1})\right) dF_{(U_{0},U_{1})}}dF_{(U_{0},U_{1})}\\{} & {} \quad \left( \int f_{0}(t,U_{0})\tfrac{\exp \left( -\varLambda ^{0}(t,U_{0})\right) }{\int \exp \left( -\varLambda ^{0}(t,U_{0})\right) dF_{U_{0}}}dF_{U_{0}}\right) ^{-1}, \end{aligned}$$

where $\varLambda ^{a}(t,u_{0},u_{1}) = \int _{0}^{t}f_{0}(s,u_{0})f_{1}(s,u_{1},a)ds$ and thus $\varLambda ^{0}(t,u_{0}) = \int _{0}^{t}f_{0}(s,u_{0})ds$.

As $U_{1}$ is degenerate,

$$\begin{aligned}&\int f_{0}(t,U_{0})\tfrac{\exp \left( -\int _{0}^{t}f_{0}(s,U_{0})f_{1}(s,a)ds\right) }{\int \exp \left( -\int _{0}^{t}f_{0}(s,U_{0})f_{1}(s,a)ds\right) dF_{U_{0}}}dF_{U_{0}}\\&\quad \left( \int f_{0}(t,U_{0})\tfrac{\exp \left( -\int _{0}^{t}f_{0}(s,U_{0})ds\right) }{\int \exp \left( -\int _{0}^{t}f_{0}(s,U_{0})ds\right) dF_{U_{0}}}dF_{U_{0}}\right) ^{-1}f_{1}(t,a)\\&\quad = \frac{{\mathbb {E}}\left[ f_{0}(t,U_{0}) \mid T^{a}{\ge } t\right] }{{\mathbb {E}}\left[ f_{0}(t,U_{0}) \mid T^{0}{\ge } t\right] }f_{1}(t,a). \end{aligned}$$

$$\square $$

### Proof of lemma 1

#### Proof

By Bayes rule, the probability density of $U_{0}$ given $T^{a}{\ge } t$, $f(u_{0} {\mid }T^{a}{\ge } t)$ equals

$$\begin{aligned} f_{U_{0}{\mid }T^{a}{\ge } t}(u_{0})&= \frac{{\mathbb {P}}(T^{a}{\ge } t \mid U_{0} = u_{0})f(u_{0})}{\int {\mathbb {P}}(T^{a}{\ge } t \mid U_{0})dF_{U_{0}}}\\&= \frac{\exp \left( -u_{0}\int _{0}^{t}\lambda _{0}(s)f_{1}(s,a) ds\right) f(u_{0})}{\int \exp \left( -U_{0}\int _{0}^{t}\lambda _{0}(s)f_{1}(s,a)\right) dF_{U_{0}}}. \end{aligned}$$

So that the Laplace transform of $U_{0}{\mid }T^{a}{\ge } t$ can be written as

$$\begin{aligned} {\mathcal {L}}_{U_{0}{\mid }T^{a}{\ge } t}(c)&= {\mathbb {E}}[\exp \left( -U_{0}c\right) \mid T^{a}{\ge } t]\\&= \int \exp \left( -U_{0}c\right) dF_{U_{0}{\mid }T^{a}{\ge } t}(u_{0})\\&= \int \exp \left( -u_{0}c\right) \frac{\exp \left( -u_{0}\int _{0}^{t}\lambda _{0}(s)f_{1}(s,a) ds\right) f(u_{0})}{\int \exp \left( -U_{0}\int _{0}^{t}\lambda _{0}(s)f_{1}(s,a)ds\right) dF_{U_{0}}}du_{0}\\&= \int \frac{\exp \left( -u_{0}(c+\int _{0}^{t}\lambda _{0}(s)f_{1}(s,a) ds)\right) f(u_{0})}{\int \exp \left( -U_{0}\int _{0}^{t}\lambda _{0}(s)f_{1}(s,a)ds\right) dF_{U_{0}}}du_{0}\\&= \frac{{\mathbb {E}}\left[ \exp \left( -U_{0}(c+\int _{0}^{t}\lambda _{0}(s)f_{1}(s,a) ds)\right) \right] }{{\mathbb {E}}\left[ \exp \left( -U_{0}\int _{0}^{t}\lambda _{0}(s)f_{1}(s,a)ds\right) \right] }\\&= \frac{{\mathcal {L}}_{U_{0}}(c+\int _{0}^{t}\lambda _{0}(s)f_{1}(s,a) ds)}{{\mathcal {L}}_{U_{0}}(\int _{0}^{t}\lambda _{0}(s)f_{1}(s,a) ds)}. \end{aligned}$$

Since for a random variable *X*, ${\mathbb {E}}[X] = -{\mathcal {L}}_{X}^{'}(0)$,

$$\begin{aligned} {\mathbb {E}}[U_{0} \mid T^{a}{\ge } t] = -{\mathcal {L}}_{U_{0}{\mid }T^{a}{\ge } t}^{'}(0) = -\frac{{\mathcal {L}}_{U_{0}}^{'}\left( \int _{0}^{t}\lambda _{0}(s)f_{1}(s,a)ds\right) }{{{\mathcal {L}}_{U_{0}}}\left( \int _{0}^{t}\lambda _{0}(s)f_{1}(s,a)ds\right) }. \end{aligned}$$

$$\square $$

### Proof of corollary 2

#### Proof

By Theorem 2,

$$\begin{aligned} \frac{\lim _{h\rightarrow 0}h^{-1}{\mathbb {P}}\left( T^{a} \in [t,t+h) \mid T^{a}{\ge } t \right) }{\lim _{h\rightarrow 0}h^{-1}{\mathbb {P}}\left( T^{0} \in [t,t+h) \mid T^{0}{\ge } t \right) } = \frac{{\mathbb {E}}\left[ f_{0}(t,U_{0})f_{1}(t,U_{1}) \mid T^{a}{\ge } t\right] }{{\mathbb {E}}\left[ f_{0}(t,U_{0}) \mid T^{0}{\ge } t\right] }. \end{aligned}$$

and equals

$$\begin{aligned}{} & {} \int f_{0}(t,U_{0})f_{1}(t,U_{1},a)\tfrac{\exp \left( -\varLambda ^{a}(t,U_{0},U_{1})\right) }{\int \exp \left( -\varLambda ^{a}(t,U_{0},U_{1})\right) dF_{(U_{0},U_{1})}}dF_{(U_{0},U_{1})}\\{} & {} \quad \left( \int f_{0}(t,U_{0})\tfrac{\exp \left( -\varLambda ^{0}(t,U_{0})\right) }{\int \exp \left( -\varLambda ^{0}(t,U_{0})\right) {dF_{U_{0}}}}{dF_{U_{0}}}\right) ^{-1}, \end{aligned}$$

where $\varLambda ^{a}(t,u_{0},u_{1}) = \int _{0}^{t}f_{0}(s,u_{0})f_{1}(s,u_{1},a)ds$ and thus $\varLambda ^{0}(t,u_{0}) = \int _{0}^{t}f_{0}(s,u_{0})ds$. As now $U_{0}$ is degenerate,

$$\begin{aligned} \int f_{1}(t,U_{1},a)\tfrac{\exp \left( -\varLambda ^{a}(t,U_{1})\right) }{\int \exp \left( -\varLambda ^{a}(t,U_{1})\right) dF_{U_{1}}} dF_{U_{1}} = {\mathbb {E}}\left[ f_{1}(t,U_{1},a) \mid T^{a}{\ge } t\right] , \end{aligned}$$

where $$\varLambda ^{a}(t,u_{1}) = \int _{0}^{t}\lambda _{0}(s)f_{1}(s,u_{1},a)ds$$. $$\square $$

## Laplace transforms

### Gamma

If $$X \sim \varGamma (k,\theta )$$, then $${\mathbb {E}}[X] = k\theta $$, $$\text {var}[X] = k\theta ^{2}$$,

$$\begin{aligned}{} & {} {\mathcal {L}}_{X}(c) = (1+\theta c)^{-k}, \\{} & {} {\mathcal {L}}^{'}_{X}(c) = -\frac{\theta k}{\theta c +1}{\mathcal {L}}_{X}(c), \end{aligned}$$

and

$$\begin{aligned} {\mathcal {L}}^{''}_{X}(c) = \frac{\theta ^{2}k(k+1)}{(\theta c +1)^{2}}{\mathcal {L}}_{X}(c). \end{aligned}$$

When $f_{\lambda }(t,U_{0i},U_{1i},a) = U_{0i}\lambda _{0}(t)f_{1}(t,a)$ and $U_{0} \sim \varGamma (k,\theta )$, by Lemma 1,

17 $$\begin{aligned} {\mathbb {E}}\left[ U_{0} \mid T^{a}{\ge } t\right] = \frac{\theta k}{\theta \varLambda ^{a}(t) +1}, \end{aligned}$$

where $\varLambda ^{a}(t) = \int _{0}^{t}\lambda _{0}(s)f_{1}(s,a)ds$. If ${\mathbb {E}}[U_{0}] = \mu$ and $\text {var}(U_{0}) = \theta _{0}$, then $k = \frac{\mu ^2}{\theta _{0}}$, $\theta = \frac{\theta _{0}}{\mu }$ and as such

18 $$\begin{aligned} {\mathbb {E}}\left[ U_{0} \mid T^{a}{\ge } t\right] = \frac{\mu }{\tfrac{\theta _{0}}{\mu }\varLambda ^{a}(t) +1}. \end{aligned}$$

In particular, when $k = \theta ^{-1}$, then ${\mathbb {E}}[U_{0}] = 1$, $\text {var}(U_{0}) = \theta _{0}$ and

19 $$\begin{aligned} {\mathbb {E}}\left[ U_{0} \mid T^{a}{\ge } t\right] = \left( \theta _{0}\varLambda ^{a}(t) +1\right) ^{-1}. \end{aligned}$$

### Inverse Gaussian

If $$X \sim \text {IG}(\mu ,\lambda )$$, then $${\mathbb {E}}[X] = \mu $$, $$\text {var}[X] = \tfrac{\mu ^3}{\lambda }$$,

$$\begin{aligned}{} & {} {\mathcal {L}}_{X}(c) = \exp \left( \frac{\lambda }{\mu }\left( 1-\sqrt{1+\frac{2\mu ^{2}c}{\lambda }}\right) \right) , \\{} & {} {\mathcal {L}}^{'}_{X}(c) = -\frac{\mu }{\sqrt{\tfrac{2\mu ^2c}{\lambda }+1}}{\mathcal {L}}_{X}(c), \end{aligned}$$

and

$$\begin{aligned} {\mathcal {L}}^{''}_{X}(c) = \left( \mu ^{2} \frac{\lambda }{\lambda + 2 \mu ^{2}c}+\frac{\mu }{\lambda (\tfrac{2\mu ^{2}c}{\lambda }+1)^{\tfrac{3}{2}}}\right) {\mathcal {L}}_{X}(c). \end{aligned}$$

When $f_{\lambda }(t,U_{0i},U_{1i},a) = U_{0i}\lambda _{0}(t)f_{1}(t,a)$ and $U_{0} \sim \text {IG}(\mu ,\lambda )$, by Lemma 1,

20 $$\begin{aligned} {\mathbb {E}}\left[ U_{0} \mid T^{a}{\ge } t\right] = \frac{\mu }{\sqrt{2 \varLambda ^{a}(t) \frac{\mu ^2}{\lambda }+1}}, \end{aligned}$$

where $\varLambda ^{a}(t) = \int _{0}^{t}\lambda _{0}(s)f_{1}(s,a)ds$. If $\text {var}(U_{0}) = \theta _{0}$, then $\lambda = \frac{\mu ^3}{\theta _{0}}$ and as such

21 $$\begin{aligned} {\mathbb {E}}\left[ U_{0} \mid T^{a}{\ge } t\right] = \frac{\mu }{\sqrt{2 \varLambda ^{a}(t) \tfrac{\theta _{0}}{\mu } +1}}. \end{aligned}$$

In particular when $\mu = 1$ and $\lambda = \theta _{0}^{-1}$, then ${\mathbb {E}}[U_{0}] = 1$, $\text {var}(U_{0}) = \theta _{0}$ and

22 $$\begin{aligned} {\mathbb {E}}\left[ U_{0} \mid T^{a}{\ge } t\right] = \left( 2 \theta _{0} \varLambda ^{a}(t)+1\right) ^{-\tfrac{1}{2}}. \end{aligned}$$

### Compound poisson

If $X \sim \text {CPoi}(\rho , \eta , \nu )$, then $X = \sum _{i=1}^{N}Y_{i}$, where $N \sim \text {Poi}(\rho )$, and $Y \sim \varGamma (\eta ,\nu )$, ${\mathbb {E}}[X] = \rho \eta \nu$, $\text {var}[X] = \rho \eta \nu ^{2}+\eta ^2\nu ^2\rho$,

$$\begin{aligned}{} & {} {\mathcal {L}}_{X}(c) = \exp \left( \rho \left( \left( \frac{\nu ^{-1}}{\nu ^{-1}+c}\right) ^{\eta }-1\right) \right) , \\{} & {} {\mathcal {L}}^{'}_{X}(c) = -\rho \eta \nu \left( \frac{1}{c \nu +1}\right) ^{\eta +1}{\mathcal {L}}_{X}(c), \end{aligned}$$

and

$$\begin{aligned} {\mathcal {L}}^{''}_{X}(c) = \eta \nu ^2 \rho \left( \frac{1}{c \nu +1}\right) ^{\eta +2} \left( \eta \rho \left( \frac{1}{c \nu +1}\right) ^{\eta }+\eta +1\right) {\mathcal {L}}_{X}(c). \end{aligned}$$

When $f_{\lambda }(t,U_{0i},U_{1i},a) = U_{0i}\lambda _{0}(t)f_{1}(t,a)$ and $U_{0} \sim \text {CPoi}(\rho , \eta , \nu )$, by Lemma 1,

23 $$\begin{aligned} {\mathbb {E}}\left[ U_{0} \mid T^{a}{\ge } t\right] = \rho \eta \nu \left( \frac{1}{\varLambda ^{a}(t) \nu +1}\right) ^{\eta +1}, \end{aligned}$$

where $\varLambda ^{a}(t) = \int _{0}^{t}\lambda _{0}(s)f_{1}(s,a)ds$. Let ${\mathbb {E}}[U_{0}] = \mu$ and $\text {var}(U_{0}) = \theta _{0}$, then $\rho = \frac{\mu ^{2}(1+\eta )}{\eta \theta _{0}}$, $\nu = \frac{\theta _{0}}{\mu (1+\eta )}$ and as such

24 $$\begin{aligned} {\mathbb {E}}\left[ U_{0} \mid T^{a}{\ge } t\right] = \mu \left( \frac{1}{\varLambda ^{a}(t) \tfrac{\theta _{0}}{\mu (1+\eta )} +1}\right) ^{\eta +1}. \end{aligned}$$

In particular, when $\eta = \tfrac{1}{2}$, $\rho = 3\theta _{0}^{-1}$ and $\nu = \tfrac{2}{3} \theta _{0},$ then ${\mathbb {E}}[U_{0}] = 1$, $\text {var}(U_{0}) = \theta _{0}$ and

25 $$\begin{aligned} {\mathbb {E}}\left[ U_{0} \mid T^{a}{\ge } t\right] = (1+\varLambda ^{a}(t)\tfrac{2}{3}\theta _{0})^{-\tfrac{3}{2}}. \end{aligned}$$

### Discrete

Let ${\mathbb {P}}(X = \mu _{i}) = p_{i}$ for $i>0$ and $i \le k$,

$$\begin{aligned}{} & {} {\mathcal {L}}_{X}(c) = \sum _{i=1}^{n} p_{i} \exp \left( -c \mu _{i} \right) , \\{} & {} {\mathcal {L}}^{'}_{X}(c) = \sum _{i=1}^{n} -\mu _{i}p_{i} \exp \left( -c \mu _{i} \right) , \end{aligned}$$

and

$$\begin{aligned} {\mathcal {L}}^{''}_{X}(c) = \sum _{i=1}^{n} \mu _{i}^{2} p_{i} \exp \left( -c \mu _{i} \right) . \end{aligned}$$

Furthermore, if $Y \sim F$, then

$$\begin{aligned}{} & {} {\mathcal {L}}_{XY}(c) = \sum _{i=1}^{n} p_{i} {\mathcal {L}}_{Y}(c\mu _{i}), \\{} & {} {\mathcal {L}}_{XY}^{'}(c) = \sum _{i=1}^{n} p_{i}\mu _{i} {\mathcal {L}}_{Y}^{'}(c\mu _{i}), \end{aligned}$$

and

$$\begin{aligned} {\mathcal {L}}_{XY}^{''}(c) = \sum _{i=1}^{n} p_{i}\mu _{i}^{2} {\mathcal {L}}_{Y}^{''}(c\mu _{i}). \end{aligned}$$

Let $U_{1}$ equal $\mu _{1}$, $\mu _{2}$ or 1 with probability $p_{1}$, $p_{2}$ and $1-p_{1}-p_{2}$ respectively. If

$$\begin{aligned} p_{2} = \frac{\left( \mu -\mu _1 p_1+p_1-1\right) {}^2}{\mu ^2-2 \mu -\mu _1^2 p_1+2 \mu _1 p_1-p_1+\theta _{1}+1}, \end{aligned}$$

and

$$\begin{aligned} \mu _{2} = \frac{\mu ^2-\mu -\mu _1^2 p_1+\mu _1 p_1+\theta _{1}}{\mu -\mu _1 p_1+p_1-1}, \end{aligned}$$

such that $p_{2} \in [0,1]$ and $\mu _{2}{\ge } 1$, then ${\mathbb {E}}[U_{1}] = \mu$ and $\text {var}(U_{1}) = \theta _{1}$.

When, $f_{\lambda }(t,U_{0i},U_{1i},a) = \lambda _{0}(t)(U_{1i})^{a}f_{1}(t,a)$, by Lemma 1, ${\mathbb {E}}\left[ U_{1} \mid T^{1}{\ge } t\right]$ equals

$$\begin{aligned} \frac{\mu _1 p_1 \exp \left( -\varLambda ^{1}(t) \mu _1\right) +\mu _2 p_2 \exp \left( -\varLambda ^{1}(t) \mu _2\right) +\left( 1-p_1-p_2\right) \exp \left( -\varLambda ^{1}(t)\right) }{p_1 \exp \left( -\varLambda ^{1}(t) \mu _1\right) +p_2 \exp \left( -\varLambda ^{1}(t) \mu _2\right) +\left( 1-p_1-p_2\right) \exp \left( -\varLambda ^{1}(t)\right) }, \end{aligned}$$

where $$\varLambda ^{1}(t) = \int _{0}^{t} \lambda _{0}(s) f_{1}(s,1) ds$$.

Furthermore, when $f_{\lambda }(t,U_{0i},U_{1i},a) = U_{0i} \lambda _{0}(t) (U_{1i})^{a} f_{1}(t,a)$, and $U_{0}$ and $U_{1}$ are independent, by Lemma 1, ${\mathbb {E}}\left[ U_{0}U_{1} \mid T^{1}{\ge } t\right]$ equals

$$\begin{aligned} \frac{p_{1}\mu _{1}{{\mathcal {L}}^{'}_{U_{0}}}(\varLambda ^{1}(t)\mu _{1})+ p_{2}\mu _{2}{{\mathcal {L}}^{'}_{U_{0}}}(\varLambda ^{1}(t)\mu _{2})+ (1-p_{1}-p_{2}){{\mathcal {L}}^{'}_{U_{0}}}(\varLambda ^{1}(t))}{p_{1}{{\mathcal {L}}_{U_{0}}}(\varLambda ^{1}(t)\mu _{1})+ p_{2}{{\mathcal {L}}_{U_{0}}}(\varLambda ^{1}(t)\mu _{2})+ (1-p_{1}-p_{2}){{\mathcal {L}}_{U_{0}}}(\varLambda ^{1}(t))}. \end{aligned}$$

## Supplementary tables

**Table 2:** Conditional expectations and resulting SMCHR when the CHR equals $\mu$ for different frailty distributions such that ${\mathbb {E}}[U_{0}] = 1$ and $\text {var}(U_{0}) = \theta _{0}$ (in absence of effect modification)

	$$U_{0}\sim $$		
	$\varGamma (\theta _{0}^{-1},\theta _{0})$	$\text {IG}(1,\theta _{0}^{-1})$	$\text {CPoi}\left( 3\theta _{0}^{-1},\tfrac{1}{2},\tfrac{2}{3}\theta _{0}\right)$
${\mathbb {E}}[U_{0} \mid T^{1}{\ge } t]$	$\frac{60}{60+\mu t^{3}\theta _{0}}$	$\sqrt{\frac{30}{30+\mu t^{3}\theta _{0}}}$	$90^{\tfrac{3}{2}}\left( 90+\mu t^3\theta _{0}\right) ^{-\tfrac{3}{2}}$
${\mathbb {E}}[U_{0} \mid T^{0}{\ge } t]$	$\frac{60}{60+t^{3}\theta _{0}}$	$\sqrt{\frac{30}{30+t^{3}\theta _{0}}}$	$90^{\tfrac{3}{2}}\left( 90+t^3\theta _{0}\right) ^{-\tfrac{3}{2}}$
$\tfrac{\lim _{h\rightarrow 0}h^{-1}{\mathbb {P}}\left( T^{1} \in (t,t+h) \mid T^{1}{\ge } t \right) }{\lim _{h\rightarrow 0}h^{-1}{\mathbb {P}}\left( T^{0} \in [t,t+h) \mid T^{0}{\ge } t \right) }$	$1+\frac{60(\mu -1)}{60+\mu t^{3}\theta _{0}}$	$\mu \frac{\sqrt{30+t^3\theta _{0}}}{\sqrt{30+\mu t^3\theta _{0}}}$	$\mu \left( \frac{90+t^3\theta _{0}}{90+\mu t^3\theta _{0}}\right) ^{\tfrac{3}{2}}$

**Table 3:** Conditional expectation of the individual effect modifier $U_{1}$ when the CHR equals $\mu$ for different modifier distributions such that ${\mathbb {E}}[U_{1}] = \mu$ and $\text {var}(U_{0}) = \theta _{1}$ (in absence of frailty)

	$$U_{1}\sim $$			
	$\varGamma (\tfrac{\mu }{\theta _{1}},\tfrac{\theta _{1}}{\mu })$	$\text {IG}\left( \mu ,\tfrac{\mu ^3}{\theta _{1}}\right)$	$\text {CPoi}\left( 3\tfrac{\mu ^2}{\theta _{1}},\tfrac{1}{2},\tfrac{2\theta _{1}}{3\mu }\right)$	$\text {BHN}(p_{1},\mu _{1},p_{2},\mu _{2})$
${\mathbb {E}}[U_{1} \mid T^{1}{\ge } t]$	$\frac{60 \mu ^{2}}{\theta _{1}t^{3}+60\mu }$	$\frac{\mu \sqrt{30\mu }}{\sqrt{ t^{3}\theta _{1}+30\mu }}$	$\mu \left( \frac{\theta _{1}t^{3}}{90\mu }+1\right) ^{-\tfrac{3}{2}}$	$\frac{p_{1}\mu _{1}+p_{2}\mu _{2}\exp \left( \tfrac{-t^3(\mu _{2}-\mu _{1})}{60}\right) +(1-p_{1}-p_{2})\exp \left( \tfrac{-t^3(1-\mu _{1})}{60}\right) }{p_{1}+p_{2}\exp \left( \tfrac{-t^3(\mu _{2}-\mu _{1})}{60}\right) +(1-p_{1}-p_{2})\exp \left( \tfrac{-t^3(1-\mu _{1})}{60}\right) }$

**Table 4:** Conditional expectation ${\mathbb {E}}[U_{0}U_{1} \mid T^{1}{\ge } t]$ and its limiting forms when $U_{1}$ follows a $\text {BHN}(p_{1},\mu _{1},p_{2},\mu _{2})$ distribution for different unit-expectations frailty distributions with $\text {var}(U_{0}) = \theta _{0}$ while . Here $p_{3} = 1-p_{1}-p_{2}$ and $\mu _{3} = 1$. For comparison ${\mathbb {E}}[U_{0} \mid T^{0}{\ge } t]$, as shown before in Table 2, is also presented

$U_{0}\sim$	${\mathbb {E}}[U_{1}U_{0} \mid T^{1}{\ge } t]$	${\mathbb {E}}[U_{0} \mid T^{0}{\ge } t]$
$\varGamma (\theta _{0}^{-1},\theta _{0})$	$\frac{\sum _{i=1}^{3} p_{i}\mu _{i}\left( 1+\tfrac{\theta _{0}t^3}{60}\mu _{i}\right) ^{-(1+\theta _{0}^{-1})}}{\sum _{i=1}^{3} p_{i}(1+\tfrac{\theta _{0}t^3}{60}\mu _{i})^{-\theta _{0}^{-1}}}$	$\frac{60}{60+t^{3}\theta _{0}}$
	$$ = (\theta _{0}\tfrac{t^3}{60})^{-1} + o\left( t^{-3}\right) $$	
$\text {IG}(1,\theta _{0}^{-1})$	$\frac{\sum _{i=1}^{3} p_{i}\mu _{i}\left( 2\theta _{0}\tfrac{t^3}{60}\mu _{i}\right) ^{-\tfrac{1}{2}}\exp \left( \theta _{0}^{-1}\left( 1-\sqrt{1+\theta _{0}2\tfrac{t^{3}}{60}\mu _{i} }\right) \right) }{\sum _{i=1}^{3} p_{i}\exp \left( \theta _{0}^{-1}\left( 1-\sqrt{1+\theta _{0}2\tfrac{t^{3}}{60}\mu _{i} }\right) \right) }$	$\sqrt{\frac{30}{30+t^{3}\theta _{0}}}$
	$$ = \sqrt{\mu _{1}}\sqrt{\tfrac{30}{t^{3}\theta _{0}}} + o\left( t^{-\tfrac{3}{2}}\right) $$	
$\text {CPoi}\left( 3\theta _{0}^{-1},\tfrac{1}{2},\tfrac{2}{3}\theta _{0}\right)$	$\frac{\sum _{i=1}^{3} p_{i}\mu _{i} \left( \frac{3}{3+2\theta _{0}\mu _{i}\tfrac{t^3}{60}}\right) ^{\tfrac{3}{2}}\exp \left( 3\theta _{0}^{-1}\sqrt{\frac{3}{3+2\theta _{0}\mu _{i}\tfrac{t^3}{60}}}-1\right) }{\sum _{i=1}^{3} p_{i}\exp \left( 3\theta _{0}^{-1}\sqrt{\frac{3}{3+2\theta _{0}\mu _{i}\tfrac{t^3}{60}}}-1\right) }$	$90^{\tfrac{3}{2}}\left( 90+t^3\theta _{0}\right) ^{-\tfrac{3}{2}}$
	$$ = 90^{\tfrac{3}{2}}\left( \theta _{0}t^{3}\right) ^{-\tfrac{3}{2}}\sum _{i=1}^{3} \frac{p_{i}}{\sqrt{\mu _{i}}} + o\left( t^{-\tfrac{9}{2}}\right) $$	

## Survival curves examples

The survival curves of $T^{0}$ and $T^{1}$ for the examples presented in this paper can be expressed in terms of the Laplace transforms presented in the previous section as shown in Table 5, where $\varLambda ^{0}(t) = \tfrac{t^{3}}{60}$. For the example where , the survival curve for $T^{1}$ is obtained empirically from simulation. For data from a RCT, $T^{a} ~\overset{d}{=}~ T \mid A{=}a$.

**Table 5:** Survival curves for $T^{0}$ and $T^{1}$ for different $\lambda _{i}^{a}(t)$ where $\varLambda ^{0}(t) = \tfrac{t^{3}}{60}$

$\lambda _{i}^{a}(t)$	$S_{T^{0}}(t)$	$S_{T^{1}}(t)$
$U_{0i}\tfrac{t^2}{20}\mu ^{a}$	${{\mathcal {L}}_{U_{0}}}(\varLambda ^{0}(t))$	${{\mathcal {L}}_{U_{0}}}(\mu \varLambda ^{0}(t))$
$\tfrac{t^2}{20}(U_{1i})^{a}$	$\exp \left( -\varLambda ^{0}(t)\right)$	${{\mathcal {L}}_{U_{1}}}(\varLambda ^{0}(t))$
$U_{0i}\tfrac{t^2}{20}(U_{1i})^{a}$	${{\mathcal {L}}_{U_{0}}}(\varLambda ^{0}(t))$	${{\mathcal {L}}_{U_{0}U_{1}}}(\varLambda ^{0}(t))$
